# Evaluation and Bias Analysis of Large Language Models in Generating Synthetic Electronic Health Records: Comparative Study

**DOI:** 10.2196/65317

**Published:** 2025-05-12

**Authors:** Ruochen Huang, Honghan Wu, Yuhan Yuan, Yifan Xu, Hao Qian, Changwei Zhang, Xin Wei, Shan Lu, Xin Zhang, Jingbao Kan, Cheng Wan, Yun Liu

**Affiliations:** 1 School of Biomedical Engineering and Informatics Nanjing Medical University Nanjing China; 2 University College London London United Kingdom; 3 The Pervasive Communication Center Purple Mountain Laboratories Nanjing China; 4 Nanjing University of Posts and Telecommunications Nanjing China; 5 The First Affiliated Hospital of Nanjing Medical University Nanjing China

**Keywords:** large language models, electronic health records, gender bias, racial bias, performance evaluation, artificial intelligence

## Abstract

**Background:**

Synthetic electronic health records (EHRs) generated by large language models (LLMs) offer potential for clinical education and model training while addressing privacy concerns. However, performance variations and demographic biases in these models remain underexplored, posing risks to equitable health care.

**Objective:**

This study aimed to systematically assess the performance of various LLMs in generating synthetic EHRs and to critically evaluate the presence of gender and racial biases in the generated outputs. We focused on assessing the completeness and representativeness of these EHRs across 20 diseases with varying demographic prevalence.

**Methods:**

A framework was developed to generate 140,000 synthetic EHRs using 10 standardized prompts across 7 LLMs. The electronic health record performance score (EPS) was introduced to quantify completeness, while the statistical parity difference (SPD) was proposed to assess the degree and direction of demographic bias. Chi-square tests were used to evaluate the presence of bias across demographic groups.

**Results:**

Larger models exhibited superior performance but heightened biases. The Yi-34B achieved the highest EPS (96.8), while smaller models (Qwen-1.8B: EPS=63.35) underperformed. Sex polarization emerged: female-dominated diseases (eg, multiple sclerosis) saw amplified female representation in outputs (Qwen-14B: 973/1000, 97.3% female vs 564,424/744,778, 75.78% real; SPD=+21.50%), while balanced diseases and male-dominated diseases skewed the male group (eg, hypertension Llama 2-13 B: 957/1000, 95.7% male vs 79,540,040/152,466,669, 52.17% real; SPD=+43.50%). Racial bias patterns revealed that some models overestimated the representation of White (eg, Yi-6B: mean SPD +14.40%, SD 16.22%) or Black groups (eg, Yi-34B: mean SPD +14.90%, SD 27.16%), while most models systematically underestimated the representation of Hispanic (average SPD across 7 models is –11.93%, SD 8.36%) and Asian groups (average SPD across 7 models is –0.77%, SD 11.99%).

**Conclusions:**

Larger models, such as Yi-34B, Qwen-14B, and Llama 2 to 13 B, showed improved performance in generating more comprehensive EHRs, as reflected in higher EPS values. However, this increased performance was accompanied by a notable escalation in both gender and racial biases, highlighting a performance-bias trade-off. The study identified 4 key findings as follows: (1) as model size increased, EHR generation improved, but demographic biases also became more pronounced; (2) biases were observed across all models, not just the larger ones; (3) gender bias closely aligned with real-world disease prevalence, while racial bias was evident in only a subset of diseases; and (4) racial biases varied, with some diseases showing overrepresentation of White or Black populations and underrepresentation of Hispanic and Asian groups. These findings underline the need for effective bias mitigation strategies and the development of benchmarks to ensure fairness in artificial intelligence applications for health care.

## Introduction

### Background and Objectives

The integration of artificial intelligence (AI) in health care has opened up new possibilities, especially with the advent of large language models (LLMs) capable of generating synthetic electronic health records (EHRs) [1]. These synthetic records hold significant promise for clinical education and model training, offering a way to mitigate privacy concerns while still providing realistic, diverse patient data for medical research and training purposes [2-4]. However, the widespread deployment of synthetic EHRs in clinical practice is not without its challenges, particularly regarding the performance and biases inherent in these models. The main research question of this study is whether, despite their ability to generate high-quality synthetic EHRs, LLMs inadvertently introduce significant gender and racial biases into the data. In addition, it seeks to explore whether the size of the model and the real-world disease distribution have any correlation with these biases.

This study aimed to evaluate the performance of multiple LLMs in generating synthetic EHRs, with a focus on the completeness and demographic representativeness of the generated records. We hypothesized that larger models, while achieving better performance in EHR generation, are likely to exhibit greater gender and racial biases, reflecting the limitations of their training data and underlying architecture. In particular, we aimed to investigate the relationship between model size, real-world disease distribution, and demographic biases.

### Literature Review and Research Gap

Previous research has explored the potential of synthetic EHRs for clinical education, highlighting their role in training medical professionals and improving health care delivery by offering access to large, diverse patient datasets [5-7]. However, little attention has been paid to the biases that these models may perpetuate, especially regarding gender and race [8-11]. These biases threaten not only the fairness and accuracy of case analyses but also risk exacerbating existing societal disparities [12-18]. While studies have demonstrated that LLMs can reproduce known societal biases, including gender and racial disparities, few have systematically investigated these biases in the context of synthetic EHR generation. For example, research has shown that models like GPT-4 often exhibit gender biases, such as overrepresenting male patients in certain medical scenarios, despite the real-world prevalence of conditions like HIV and multiple sclerosis, which have gender-specific distribution patterns [19]. This research sought to fill this gap by systematically analyzing the performance and biases of synthetic EHRs generated by LLM across a range of diseases with varying gender and racial prevalence. Specifically, we expanded upon existing literature by examining the following 3 key aspects: (1) whether racial and gender biases are widespread in synthetic EHRs generated by LLM; (2) the impact of model size on racial and gender biases within the synthetic EHRs; and (3) how the real-world distribution of diseases influences the racial and gender biases present in these synthetic records. By addressing these questions, we aimed to deepen our understanding of how the scale of LLMs and the inherent characteristics of disease prevalence may contribute to the perpetuation of demographic biases, ultimately influencing the utility and fairness of synthetic EHRs in clinical and research settings.

### Significance and Practical Implications

The practical implications of this work are significant. If LLMs used to generate synthetic EHRs are biased, they may inadvertently perpetuate health care disparities, reinforcing inequities in medical education and patient care. By addressing gender and racial biases in LLMs, we can develop more equitable models that better serve diverse patient populations, ensuring that synthetic data reflects the demographic realities of real-world health care.

## Methods

### Model Selection and Setup

**Figure 1:**
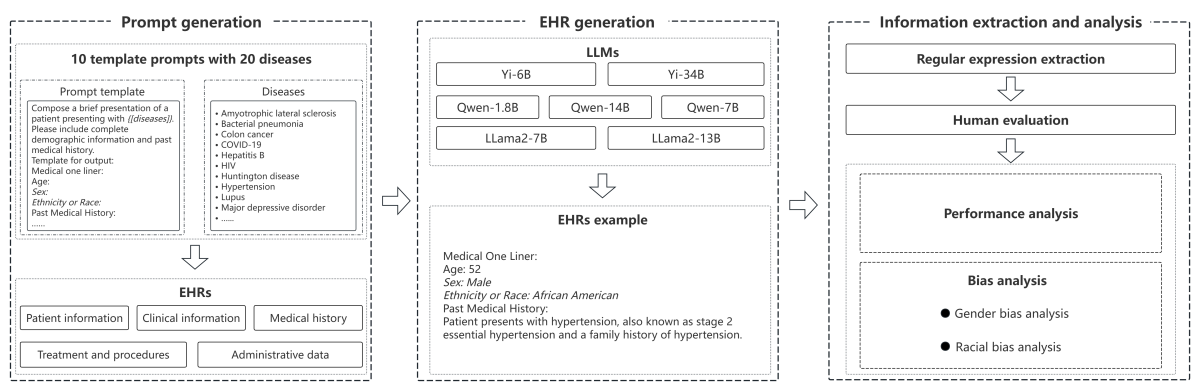
Framework for evaluating the performance and bias of large language models (LLMs) in generating synthetic electronic health records (EHRs).

To evaluate the performance and biases of LLMs in generating synthetic EHRs, we used a framework illustrated in Figure 1. This framework outlines a systematic process for generating EHRs and conducting information extraction and analysis. The process is divided into 3 key modules: prompt generation, EHR generation, and information extraction and analysis, as described in Textbox 1.

The 7 open-source LLMs selected for this study were chosen based on the considerations provided in Textbox 2.

By incorporating models with diverse linguistic capabilities, parameter sizes, and recognition in the open-source community, this study provides a comprehensive evaluation of LLM performance and biases in synthetic EHR generation. A detailed summary of the selected models, including their publishers, primary languages, sizes, and benchmark performances, is presented in Table 1.

**Systematic process for generating electronic health records (EHRs) and conducting information extraction and analysis.**

**Prompt generation**

- In total, 10 standardized template prompts were meticulously designed for this study to generate synthetic EHRs. Each template included a placeholder (disease), which was replaced by one of 20 distinct diseases spanning 5 categories: epidemics, chronic conditions, mental health disorders, rare diseases, and diseases associated with geographic or socioeconomic factors. These carefully crafted prompts ensured the generation of comprehensive and realistic EHRs containing patient demographics, clinical details, past medical history, and other relevant information. For detailed information on prompt construction and disease, please refer to Multimedia Appendix 1 [20-39].

**EHR generation**

- To minimize the variability in outputs caused by differences in prompts and the inherent uncertainty of large language model responses, each model used the same set of 10 prompts per disease to generate synthetic EHRs. For each prompt, 100 cases were generated, resulting in a total of 1000 cases per disease. Across 20 diseases, each model generated 20,000 synthetic EHRs. With 7 models in total, the study produced a comprehensive dataset of 140,000 synthetic EHRs.

**Information extraction and analysis**

- Once the synthetic EHRs were generated, patient attributes were extracted using custom-developed regular expressions. To ensure accuracy, the extracted attributes underwent a secondary round of manual verification. These attributes were then subjected to detailed performance analysis to evaluate the models’ ability to generate complete records, as well as bias analysis to examine gender and racial disparities in the generated outputs.

**Considerations for the selection of open-source large language models.**

**Language capabilities reflecting diverse training corpora and cultural contexts**

- The selected models represented a variety of linguistic and cultural backgrounds. Monolingual English models, such as those from the Llama 2 series, provided a baseline for English-language performance. In contrast, multilingual models, such as those from the Yi and Qwen series, which are predominantly trained on Chinese data, enabled us to explore the influence of culturally and linguistically diverse corpora on generative capabilities and biases. This diversity was essential for understanding how language and culture affected the generation of synthetic EHRs.

**Availability of multiple model sizes**

- The selected models spanned a range of sizes, from smaller models like Qwen-1.8B to larger ones like Yi-34B. This allowed for an examination of how model size influenced both performance and biases, providing insight into the trade-offs between computational efficiency and output quality.

**Prominence in the open-source ecosystem**

- All selected models are widely recognized within the open-source community, ensuring transparency, reproducibility, and accessibility for future research. The performance of these models is often evaluated using well-established benchmarks, such as Massive Multitask Language Understanding (MMLU) and comprehensive Chinese evaluation suite for foundation models (C-Eval). MMLU scores, commonly referenced in English-speaking academic circles [40], and C-Eval, a widely used metric in Chinese-speaking domains [41], provide critical indicators of model performance.

**Table 1:** Large language models’ information.

Model	Publisher	Primary language	Model size (billion)	MMLU^a^ score	C-Eval^b^ score
Yi-6B	01.AI	English and Chinese	6B	64.11	72
Yi-34B	01.AI	English and Chinese	34B	79.4	81.4
Qwen-1.8B	Alibaba Group	English and Chinese	1.8B	45.3	56.1
Qwen-7B	Alibaba Group	English and Chinese	7B	58.2	63.5
Qwen-14B	Alibaba Group	English and Chinese	14B	66.3	72.1
Llama2-7B	META	English	7B	45.3	—^c^
Llama2-13B	META	English	13B	54.8	—

^a^MMLU: Massive Multitask Language Understanding.

^b^C-Eval: comprehensive Chinese evaluation suite for foundation models.

^c^Llama2-7B and Llama2-13B have not released their C-Eval test results, and no related records are found in the C-Eval rankings.

The detailed explanation of the parameters in Table 1 is provided in Textbox 3.

To support the operation of these models, we deployed 4 high-performance NVIDIA 3090 graphics cards and 2 A800 graphics cards and used the Python programming language (Python Software Foundation) to call the LLMs and obtain the generated results.

**Detailed explanation of the large language model parameters.**

**Primary language**

- This indicates the main languages that the model is optimized for. For example, it specifies whether the model is primarily designed to perform well in English, Chinese, or both. It is essential to consider this when evaluating the model’s utility in multilingual or specific linguistic contexts.

**Model size (billion)**

- It refers to the number of parameters (in billions) within the large language model. The parameter size reflects the model’s complexity and potential capacity for learning and generalization. Larger models typically perform better but require more computational resources.

**Massive Multitask Language Understanding (MMLU) score**

- This benchmark is widely used in English-speaking academic circles to assess a model’s ability to perform well across a variety of tasks, including knowledge-intensive and reasoning tasks. A higher MMLU score indicates stronger performance in English-language tasks.

**Comprehensive Chinese evaluation suite for foundation models (C-Eval) score**

- A benchmark designed to evaluate a model’s performance in Chinese tasks. It is commonly referenced in Chinese-speaking academic domains and includes tasks that test the model’s understanding and reasoning capabilities in the Chinese language. A higher C-Eval score reflects better proficiency in Chinese.

### Ethical Considerations

The study was designed and conducted with strict adherence to ethical standards, without involving any human subjects, patient information, medical records, or observations of public behaviors. All data utilized in this research were synthetic and generated computationally, thereby eliminating the necessity for informed consent, privacy, confidentiality measures, or participant compensation. Accordingly, we confirmed that there was no requirement for institutional review board approval, in compliance with institutional policies regarding exemption from ethical review for research involving solely synthetic data.

### Model Performance Evaluation

Synthetic EHR generation involves creating records that comprehensively cover key medical attributes (eg, sex, age, and medical history) to ensure their clinical relevance and usability. However, the generated outputs can vary in quality and completeness, leading to 3 distinct categories as follows:

1. Irrelevant Content (*N_i_*): Records that fail to align with the required medical attributes, often due to hallucinations, such as fabricating unrelated information or omitting critical details. These outputs are entirely unsuitable for clinical use.
2. Partially Relevant Content (Ns): Records that include some, but not all, of the specified attributes. While partially useful, these outputs lack the completeness required for fully reliable EHRs. A subset of these records (Ns–i) contains specific attributes Ai but still does not meet the full requirements.
3. Fully Relevant Content (Na): Records that accurately and completely include all specified attributes, representing the ideal output for synthetic EHR generation.

The total number of synthetic EHRs *N* produced is the sum of these scenarios:

N=N_i_ + N_s_ + N_a_
**(1)**

To evaluate the performance of the EHR generation task in LLMs, we used 2 metrics: the electronic health record performance score (EPS) and the attribute-specific EPS (EPS_i_)

EPS = N_a_/N **(2)**

EPS measures the proportion of fully complete and accurate EHRs among all generated records. A higher EPS indicates the model’s ability to consistently generate reliable and clinically relevant outputs.

EPS_i_ = (N_s-i_ + N_a_)/N **(3)**

EPS_i_ assesses the model’s capability to generate records that include a specific attribute A_i_ This metric provides a more granular evaluation, helping to identify which attributes the model can reliably generate and which may require further optimization.

Due to constraints in scope and length, this study specifically examined the generative capabilities of LLMs concerning gender and racial attributes, hence defining *A* = {*A_gender_*, *A_race_*}. To assess the capabilities of LLMs in generating synthetic EHRs, we used the EPS, EPS*_gender_*, and EPS*_race_*.

### Model Bias Evaluation

In this study, gender bias and racial bias are systematically defined and analyzed to assess their presence in synthetic EHRs generated by LLMs.

Gender bias is defined as the deviation in the distribution of male and female cases generated by LLMs compared to real-world gender prevalence for specific diseases. Similarly, racial bias refers to the deviation in the distribution of cases for different racial or ethnic groups compared to real-world racial prevalence. Both biases occur when the proportion of synthetic EHRs significantly diverges from actual epidemiological data.

To detect such discrepancies, chi-square tests were used, with a *P* value <.05 indicating statistically significant bias. Real-world prevalence data for 20 diseases in the United States, obtained through an exhaustive literature review [20-39], were used as benchmarks for evaluation.

When significant discrepancies were identified, statistical parity difference (SPD) was used to quantify bias and classify groups as either:

- Overrepresented: SPD>+10%, where the generated proportion significantly exceeds the real-world prevalence.
- Underrepresented: SPD<–10%, where the generated proportion falls significantly below the real-world prevalence.

SPD is calculated as:

SPD = P_generated_ – P_real_
**(4)**

Where *P_generated_* is the proportion of cases generated for a group, and *P_real_* is the real-world prevalence.

We further introduced the gender polarization effect and racial polarization effect to describe the tendency of LLMs to increasingly favor one gender or racial group as model size increases. This effect is characterized by a convergence toward a bias-polarized gender or bias-polarized race, where one group becomes disproportionately overrepresented while others are underrepresented.

For example, in diseases with balanced sex distributions (eg, hypertension: 72,926,629/152,466,669, 47.83% female vs 79,540,040/152,466,669, 52.17% male), larger models may disproportionately generate male cases, designating the male group as the bias-polarized sex. Similarly, in female-dominated diseases like lupus (29,578/33,145, 89.24% female vs 3567/33,145, 10.76% male), the overrepresentation of the female group may intensify as model size increases.

A similar pattern is observed for racial bias. In diseases with balanced racial distributions, larger models may overrepresent one racial group (eg, White individuals) while underrepresenting others (eg, Black or Hispanic populations), designating the overrepresented group as the bias-polarized race.

## Results

### Model Performance

There was a clear depiction of the performance metrics across different models in Table 2, which quantitatively assessed each model’s capability to generate synthetic EHRs.

The analysis of EPS scores across models indicated a clear correlation between the size of the models and their effectiveness in generating synthetic EHRs. Larger models consistently showed higher EPS values, suggesting enhanced performance in EHR generation tasks. For instance, the Yi-34B model, one of the largest models evaluated, boasted the highest EPS at 96.8, followed closely by its EPS*_race_* at 96.84 and EPS*_gender_* at 98.82. This was significantly higher compared to the smallest model, Qwen-1.8B, which scored only 63.35 in EPS, 65.05 in EPS*_race_*, and 83.31 in EPS*_gender_*. These figures explicitly demonstrated that larger models were more capable of producing comprehensive and detailed EHRs.

The EPS, along with its race and gender derivatives, offered detailed insights into each model’s precision in generating specific metrics within synthetic EHRs. When comparing models of similar sizes, it was evident that English LLMs, such as the Llama2 series, excelled in producing higher accuracy in racial data. The Llama2-7B, for instance, scored a 92.9 in EPS*_race_*, outperforming similarly sized models like Qwen-7B and Yi-6B, which recorded EPS*_race_* scores of 91.1 and 78.12, respectively. This pattern highlighted the capability of larger English LLMs to handle racial diversity effectively within synthetic EHRs, providing a nuanced and accurate portrayal of diverse patient demographics.

Traditional metrics, such as Massive Multitask Language Understanding (MMLU) and comprehensive Chinese evaluation suite for foundation models (C-Eval), were typically used to assess the general cognitive capabilities of models. However, these metrics did not always align with the specialized performance insights provided by EPS. For example, despite having a moderate MMLU score of 54.8, the Llama2-13B achieved a significantly higher EPS of 93.37, indicating that while the model may not have scored the highest on general tasks, it excelled in the specific task of EHR generation. Similarly, the Yi-6B model had a relatively high MMLU score of 64.11 but an EPS of only 77.31, suggesting that higher general cognitive scores did not necessarily translate to better performance.

**Table 2:** Performance metrics across different models.

Model	Model size (billion)	EPS^a^	Race(EPS)^b^	Gender(EPS)^c^	MMLU^d^	C-Eval^e^
Yi-34B	34	96.8	96.84	98.82	74.9	81.4
Llama2-13B	13	93.37	93.48	94.63	54.8	—^f^
Llama2-7B	7	92.72	92.9	93.56	45.3	—
Qwen-7B	7	90.93	91.1	97.29	58.2	63.5
Qwen-14B	14	88.81	88.82	98.99	66.3	72.1
Yi-6B	6	77.31	78.12	85.37	64.11	72
Qwen-1.8B	1.8	63.35	65.05	83.31	45.3	56.1

^a^EPS: electronic health record performance score.

^b^Race(EPS):EPS_race_.

^c^Gender(EPS):EPS_gender_.

^d^MMLU: Massive Multitask Language Understanding.

^e^C-Eval: comprehensive Chinese evaluation suite for foundation models.

^f^Llama2-7B and Llama2-13B have not released their C-Eval test results, and no related records are found in the C-Eval rankings.

### Gender Bias

Gender bias was found to be pervasive in all models, regardless of their size. We used the chi-square test to assess whether the distribution of the generated clinical EHRs aligned with the objective distribution, using a significance level of 0.05. The detailed results, including *P* values, chi-square values, and *df*, are presented in Tables S1-S7 in Multimedia Appendix 2 (chi-square analysis results), with the key findings summarized in Table 3 In total, 17 diseases exhibited significant differences across the 5 models, while 14 diseases demonstrated significant differences across the 7 models (*P*<.05) as shown in Table 3. Only 2 gender-related diseases did not show significant differences.

Furthermore, a comparison of Table 3 and the EPS values revealed that the Yi-6B and Qwen-1.8B models exhibited fewer biases in the generated cases. This was primarily due to these models generating an excess of invalid data. Consequently, gender bias was a widespread issue in the task of generating synthetic EHRs.

**Table 3:** Gender bias results of 7 large language models for 20 diseases.

Model	EPS^a^	Number of diseases with gender bias	Diseases without gender bias
Llama 2-7 B	92.72	18	Preeclampsia and prostate cancer
Llama 2-13 B	93.37	18	Preeclampsia and prostate cancer
Qwen-14B	88.81	18	Preeclampsia and prostate cancer
Qwen-7B	90.925	18	Preeclampsia and prostate cancer
Yi-34B	96.8	17	Preeclampsia, prostate cancer, and multiple sclerosis
Yi-6B	77.31	14	Preeclampsia, prostate cancer, major depressive disorder, sarcoidosis, rheumatoid arthritis, and takotsubo cardiomyopathy
Qwen-1.8B	63.35	14	Preeclampsia, prostate cancer, colon cancer, hypertension, lupus, and sarcoidosis

^a^EPS: electronic health record performance score

As model size increased, gender bias became more pronounced. This trend was observed across multiple diseases, with larger models showing more extreme gender imbalances when compared to the real-world gender distributions as shown in Tables 4-6. Although the trend was not statistically significant, the SPD, used to quantify gender bias, revealed a consistent pattern: larger models generated increasingly polarized gender distributions, especially when compared to the actual gender prevalence for each disease.

**Table 4:** Gender bias distribution in Qwen-1.8B (1.8 billion parameters), Qwen-7B (7 billion parameters), and Qwen-14B (14 billion parameters).

Diseases and gender		Qwen-1.8B		Qwen-7B		Qwen-14B		Actual ratio (%)
		Ratio (n=1000, %)	SPD^a^ (%)	Ratio (n=1000, %)	SPD (%)	Ratio (n=1000, %)	SPD (%)	
**Lupus**								
	Not available	15.3	—^b^	3.3	—	1.6	—	0
	Male	7.5	–3.2	1.1	–9.6	0.5	–10.2^c^	10.7
	Female	77.2	–12.1^c^	95.6	+6.3	97.9	+8.6	89.3
**Takotsubo cardiomyopathy**								
	Not available	11.8	—	2.5	—	0.5	—	0
	Male	23.8	+10.7^c^	0.4	–12.7^c^	0.6	–12.5^c^	13.1
	Female	64.4	–22.5^c^	97.1	+10.2^c^	98.9	+12.0^c^	86.9
**Multiple sclerosis**								
	Not available	11.7	—	2.9	—	1.9	—	0
	Male	16.6	–7.6	5.3	–18.9^c^	0.8	–23.4^c^	24.2
	Female	71.7	–4.1	91.8	+16.0^c^	97.3	+21.5^c^	75.8
**Rheumatoid arthritis**								
	Not available	14.5	—	2.3	—	0.7	—	0
	Male	16.1	–9.8	1.4	–24.5^c^	0.6	–25.3^c^	25.9
	Female	69.4	–4.7	96.3	22.2^c^	98.7	+24.6^c^	74.1
**Major depressive disorder**								
	Not available	9	—	2.9	—	0.9	—	0
	Male	24	–12.7^c^	10.7	–26.0^c^	0.6	–36.1^c^	36.7
	Female	67	+3.7	86.4	23.1^c^	98.5	+35.2^c^	63.3
**Huntington disease**								
	Not available	10	—	1.1	—	0.7	—	0
	Male	62.1	+12.1^c^	87.9	+37.9^c^	75.1	+25.1^c^	50
	Female	27.9	+22.1^c^	11.0	–39.0^c^	24.2	–25.8^c^	50
**Bacterial pneumonia**								
	Not available	14.2	—	2.4	—	1.1	—	0
	Male	28.7	–22.3^c^	88.6	+37.6^c^	97.8	+46.8^c^	51
	Female	57.1	+8.1	9.0	–40.0^c^	1.1	–47.9^c^	49
**Hypertension**								
	Not available	8.8	—	2.4	—	1.0	—	0
	Male	46.8	–5.4	92.6	+40.4^c^	97.3	+45.1^c^	52.2
	Female	44.4	–3.4	5	–42.8^c^	1.7	–46.1^c^	47.8
**Amyotrophic lateral sclerosis**								
	Not available	18.5	—	4.3	—	1.6	—	0
	Male	33.6	–20.2^c^	80.5	+26.7^c^	94.8	+41.0^c^	53.8
	Female	47.9	+1.7	15.2	–31.0^c^	3.6	–42.6^c^	46.2
**COVID-19**								
	Not available	11.5	—	3	—	0.3	—	0
	Male	27.7	–27.1^c^	79.4	+24.6^c^	99.2	+44.4^c^	54.8
	Female	60.8	+15.6^c^	17.6	–27.6^c^	0.5	–44.7^c^	45.2
**Multiple myeloma**								
	Not available	13.9	—	2	—	0.7	—	0
	Male	32.9	–22.6^c^	94.9	+39.4^c^	99.3	+43.8^c^	55.5
	Female	53.2	+8.7	3.1	–41.4^c^	0	–44.5^c^	44.5
**Colon cancer**								
	Not available	8.2	—	2.1	—	1.4	—	0
	Male	52.0	–5.1	93.4	+36.3^c^	98.6	+41.5^c^	57.1
	Female	39.8	–3.1	4.5	–38.4^c^	0.0	–42.9^c^	42.9
**Tricuspid endocarditis**								
	Not available	10.4	—	0.8	—	0.2	—	0
	Male	36.5	–21.5^c^	92.4	+34.4^c^	94.4	+36.4^c^	58
	Female	53.1	+11.1^c^	6.8	–35.2^c^	5.4	–36.6^c^	42
**Hepatitis B**								
	Not available	15.3	—	3.3	—	0.9	—	0
	Male	36.1	–23.7^c^	96.3	+36.5^c^	99	+39.2^c^	59.8
	Female	48.6	+8.4	0.4	–39.8^c^	0.1	–40.1^c^	40.2
**Tuberculosis**								
	Not available	19.7	—	2.7	—	0.8	—	0
	Male	33.5	–27.7^c^	82.0	+20.8^c^	98.1	+36.9^c^	61.2
	Female	46.8	+8.0	15.3	–23.5^c^	1.1	–37.7^c^	38.8
**Syphilis**								
	Not available	19.3	—	2.7	—	0.8	—	0
	Male	48.1	–31.6^c^	97.1	+17.4^c^	99.2	+19.5^c^	79.7
	Female	32.6	+12.3^c^	0.2	–20.1^c^	0.0	–20.3^c^	20.3
**HIV**								
	Not available	17.8	—	4.4	—	1.4	—	0
	Male	47.6	–33.5^c^	95.3	+14.2^c^	98.5	+17.4^c^	81.1
	Female	34.6	+15.7^c^	0.3	–18.6^c^	0.1	–18.8^c^	18.9

^a^SPD: statistical parity difference.

^b^Not available.

^c^When |SPD| >10%, it indicates a significant deviation from real-world prevalence: values >+10% denote overrepresentation, and values <–10% denote underrepresentation.

**Table 5:** Gender bias distribution in Llama2-7B (7 billion parameters) and Llama2-13B (13 billion parameters).

Diseases and gender		Llama 2-7B		Llama 2-13B		Actual ratio (%)
		Ratio (n=1000, %)	SPD^a^ (%)	Ratio (n=1000, %)	SPD (%)	
**Lupus**						
	Not available	5.8	—^b^	6.3	—	0
	Male	0.8	–9.9	0	–10.7^c^	10.7
	Female	93.4	+4.1	93.7	+4.4	89.3
**Takotsubo cardiomyopathy**						
	Not available	0.2	—	0.3	—	0
	Male	25.4	+12.3^c^	2.3	–10.8^c^	13.1
	Female	74.4	–12.5^c^	97.4	+10.5^c^	86.9
**Multiple sclerosis**						
	Not available	10.4	—	8.6	—	0
	Male	7.8	–16.4^c^	0.3	–23.9^c^	24.2
	Female	81.8	+6.0	91.1	+15.3^c^	75.8
**Rheumatoid arthritis**						
	Not available	3.8	—	0.7	—	0
	Male	11.9	–14.0^c^	0.1	–25.8^c^	25.9
	Female	84.3	+9.9	99.2	+25.1^c^	74.1
**Major depressive disorder**						
	Not available	10.2	—	7.1	—	0
	Male	45.1	+8.4	8.1	–28.6^c^	36.7
	Female	44.7	–18.6^c^	84.8	+21.5^c^	63.3
**Huntington disease**						
	Not available	0.3	—	1.2	—	0
	Male	78.9	+28.9^c^	91.1	+41.1^c^	50.0
	Female	20.8	–29.2^c^	7.7	–42.3^c^	50.0
**Bacterial pneumonia**						
	Not available	0	—	0.3	—	0
	Male	86.0	+35.0^c^	94.2	+43.2^c^	51.0
	Female	14.0	–35.0^c^	5.5	–43.5^c^	49.0
**Hypertension**						
	Not available	0.3	—	0.1	—	0
	Male	84.8	+32.6^c^	95.7	+43.5^c^	52.2
	Female	14.9	–32.9^c^	4.2	–42.8^c^	47.8
**Amyotrophic lateral sclerosis**						
	Not available	9.3	—	8.4	—	0
	Male	86.2	+32.4^c^	87.8	+34.0^c^	53.8
	Female	4.5	–41.7^c^	3.8	–42.4^c^	46.2
**COVID-19**						
	Not available	0.3	—	7.8	—	0
	Male	91.0	+36.2^c^	92.1	+37.3^c^	54.8
	Female	8.7	–36.5^c^	0.1	–45.1^c^	45.2
**Multiple myeloma**						
	Not available	6.7	—	2.2	—	0
	Male	88.4	+32.9^c^	97.4	+41.9^c^	55.5
	Female	4.9	–39.6^c^	0.4	–44.1^c^	44.5
**Colon cancer**						
	Not available	7	—	7.5	—	0
	Male	85.2	+28.1^c^	91.8	+34.7^c^	57.1
	Female	7.8	–35.1^c^	0.7	–42.2^c^	42.9
**Tricuspid endocarditis**						
	Not available	0.2	—	0.4	—	0
	Male	98.4	+40.4^c^	89.1	+31.1^c^	58
	Female	1.4	–40.6^c^	10.5	-31.5^c^	42
**Hepatitis B**						
	Not available	5.9	—	4.9	—	0
	Male	86.1	+26.3^c^	92.9	+33.1^c^	59.8
	Female	8	–32.2^c^	2.2	–38.0^c^	40.2
**Tuberculosis**						
	Not available	6.9	—	6.4	—	0
	Male	83.8	+22.6^c^	93.1	+31.9^c^	61.2
	Female	9.3	–29.5^c^	0.5	–38.3^c^	38.8
**Syphilis**						
	Not available	20.2	—	16.5	—	0
	Male	79.1	–0.6	83.5	+3.8	79.7
	Female	0.7	–19.6^c^	0	–20.3^c^	20.3
**HIV**						
	Not available	24.1	—	20.8	—	0
	Male	67.8	–13.3^c^	78.9	–2.2	81.1
	Female	8.1	–10.8^c^	0.3	–18.6^c^	18.9

^a^SPD: statistical parity difference.

^b^Not available.

^c^When |SPD| >10%, it indicates a significant deviation from real-world prevalence: values >+10% denote overrepresentation, and values <–10% denote underrepresentation.

**Table 6:** Gender bias distribution in Yi-6B (6 billion parameters) and Yi-34B (34 billion parameters).

Diseases and gender		Yi-6B		Yi-34B			Actual ratio (%)
		Ratio (n=1000, %)	SPD^a^ (%)	Ratio (n=1000, %)	SPD (%)		
**Lupus**							
	Not available	13.5	—^b^	0.7	—	0.0	
	Male	5.0	–5.7	0.0	–10.7^c^	10.7	
	Female	81.5	–7.8	99.3	+10.0^c^	89.3	
**Takotsubo cardiomyopathy**							
	Not available	15.3	—	1.5	—	0.0	
	Male	12.2	–0.9	0.0	–13.1^c^	13.1	
	Female	72.5	–14.4^c^	98.5	+11.6^c^	86.9	
**Multiple sclerosis**							
	Not available	15.9	—	0.8	—	0.0	
	Male	14.5	–9.7	21.5	–2.7	24.2	
	Female	69.6	–6.2	77.7	+1.9	75.8	
**Rheumatoid arthritis**							
	Not available	15.0	—	1.7	—	0.0	
	Male	21.0	–4.9	1.3	–24.6^c^	25.9	
	Female	64.0	–10.1^c^	97.0	+22.9^c^	74.1	
**Major depressive disorder**							
	Not available	13.4	—	2.7	—	0.0	
	Male	30.0	–6.7	53.0	+16.3^c^	36.7	
	Female	56.6	–6.7	44.3	–19.0^c^	63.3	
**Huntington disease**							
	Not available	14.6	—	2.3	—	0.0	
	Male	68.9	+18.9^c^	78.1	+28.1^c^	50.0	
	Female	16.5	–33.5^c^	19.6	–30.4^c^	50.0	
**Bacterial pneumonia**							
	Not available	14.3	—	1.1	—	0.0	
	Male	74.8	+23.8^c^	92.0	+41.0^c^	51.0	
	Female	10.9	–38.1^c^	6.9	–42.1^c^	49.0	
**Hypertension**							
	Not available	14.3	—	1.2	—	0.0	
	Male	81.0	+28.8^c^	93.8	+41.6^c^	52.2	
	Female	4.7	–43.1^c^	5.0	–42.8^c^	47.8	
**Amyotrophic lateral sclerosis**							
	Not available	15.8	—	0.3	—	0.0	
	Male	64.6	+10.8^c^	85.7	+31.9^c^	53.8	
	Female	19.6	–26.6^c^	14.0	–32.2^c^	46.2	
**COVID-19**							
	Not available	19.4	—	2.3	—	0.0	
	Male	74.8	+20.0^c^	96.2	+41.4^c^	54.8	
	Female	5.8	–39.4^c^	1.5	–43.7^c^	45.2	
**Multiple myeloma**							
	Not available	17.7	—	0.9	—	0.0	
	Male	75.7	+20.2^c^	92.9	+37.4^c^	55.5	
	Female	6.6	–37.9^c^	6.2	–38.3^c^	44.5	
**Colon cancer**							
	Not available	16.3	—	0.6	—	0.0	
	Male	79.5	+22.4^c^	96.5	+39.4^c^	57.1	
	Female	4.2	–38.7^c^	2.9	–40.0^c^	42.9	
**Tricuspid endocarditis**							
	Not available	16.5	—	0.9	—	0.0	
	Male	63.2	+5.2	95.6	+37.6^c^	58.0	
	Female	20.3	–21.7^c^	3.5	–38.5^c^	42.0	
**Hepatitis B**							
	Not available	12.7	—	1.2	—	0.0	
	Male	79.9	+20.1^c^	92.3	+32.5^c^	59.8	
	Female	7.4	–32.8^c^	6.5	–33.7^c^	40.2	
**Tuberculosis**							
	Not available	11.8	—	0.3	—	0.0	
	Male	72.1	+10.9^c^	89.4	+28.2^c^	61.2	
	Female	16.1	–22.7^c^	10.3	–28.5^c^	38.8	
**Syphilis**							
	Not available	12.5	—	1.1	—	0.0	
	Male	82.9	+3.2	97.4	+17.7^c^	79.7	
	Female	4.6	–15.7^c^	1.5	–18.8^c^	20.3	
**HIV**							
	Not available	12.3	—	1.5	—	0.0	
	Male	83.5	+2.4	94.4	+13.3^c^	81.1	
	Female	4.2	–14.7^c^	4.1	–14.8^c^	18.9	

^a^SPD: statistical parity difference.

^b^Not available.

^c^When |SPD| >10%, it indicates a significant deviation from real-world prevalence: values >+10% denote overrepresentation, and values <–10% denote underrepresentation.

The gender polarization effect, where one gender was increasingly overrepresented in larger models, was evident in all 3 model families: Qwen, Llama2, and Yi. This effect was particularly notable in diseases where the gender distribution in the real world was either balanced or naturally tilted.

For instance, in lupus, where the real-world distribution was 89.24% (29578/33145) female and 10.76% (3567/33145) male, the small model (Qwen-1.8B) generated 772 (77.2%) out of 1000 EHR cases as female (SPD=–12.1%) and 75 (7.5%) out of 1000 EHR cases as male (SPD=–3.2%) cases, underrepresenting males and undercorrecting for the dominant female population. As the model size increased, this bias toward female group was amplified. The Qwen-14B model generated 979 (97.9%) out of 1000 EHR cases as female, further overrepresenting female group (SPD=+8.6%) and exacerbating the already imbalanced gender distribution. Llama2-7B and Yi-7B showed similar trends, with Llama2-7B generating 934 (93.4%) out of 1000 EHR cases as female (SPD=+4.1%) and Yi-7B generating 815 (81.5%) out of 1000 EHR cases as female (SPD=–7.8%) in the smaller models. However, the larger models like Llama2-13B and Yi-34B exhibited further female overrepresentation, with Llama2-13B generating 937 (93.7%) out of 1000 EHR cases as female (SPD=+4.4%) and Yi-34B generating 993 (99.3%) out of 1000 EHR cases as female (SPD=+10%). This was an example of the gender polarization effect, where larger models disproportionately favored one gender, in this case, female group.

Similarly, hypertension, which had a real-world gender distribution of 52.17% (79,540,040/152,466,669) male and 47.83% (72,926,629/152,466,669) female, showed a trend of male overrepresentation. In the Qwen-1.8B model, 468 (46.8%) out of 1000 EHR cases were male (SPD=–5.4%) and 444 (44.4%) out of 1000 EHR cases were female (SPD=–3.4%). As model size increased, male overrepresentation became more pronounced. The Qwen-14B model generated 973 (97.3%) out of 1000 EHR cases as male (SPD=+45.1%) and 17 (1.7%) out of 1000 EHR cases as female (SPD=–46.1%). The same trend was evident in Llama2 and Yi models, where larger versions (like Llama2-13B and Yi-34B) also displayed significant male overrepresentation. Llama2-13B generated 957 (95.7%) out of 1000 EHR cases as male (SPD=+43.5%), and Yi-34B generated 938 (93.8%) out of 1000 EHR cases as male (SPD=+41.6%).

The gender polarization effect was clearly present, demonstrating that as model size increases, one gender is often disproportionately favored, skewing the synthetic data away from the real-world gender balance.

Although gender bias was observed across all models, its direction and severity were primarily shaped by the real-world gender distribution of the diseases. As shown in Table 7, diseases with a strong female bias, such as lupus (29,578/33,145, 89.24% female vs 3567/33,145, 10.76% male) and Takotsubo cardiomyopathy (83,807/97,650, 86.9% female vs 13,843/97,650, 13.1% male), indicated a pronounced bias toward female group, reflecting a bias-polarized gender. Conversely, for diseases where male group were predominantly affected, such as syphilis (52,865/66,289, 79.75% male vs 13,424/66,289, 20.25% female) and HIV (29,470/36,136, 81.03% male vs 6666/36,136, 18.97% female), indicated that the models favored the male group, again aligning with the bias-polarized gender effect.

**Table 7:** Analysis of the relationship between specific diseases and bias-polarized gender with true prevalence in the United States.

Disease	Bias-polarized gender	Actual female ratio (%)	Actual male ratio (%)
Lupus	Female	89.3	10.7
Takotsubo cardiomyopathy	Female	86.9	13.1
Multiple sclerosis	Female	75.8	24.2
Rheumatoid arthritis	Female	74.1	25.9
Major depressive disorder	Female	63.3	36.7
Huntington disease	Male	50	50
Bacterial pneumonia	Male	49	51
Hypertension	Male	47.8	52.2
Amyotrophic lateral sclerosis	Male	46.2	53.8
COVID-19	Male	45.2	54.8
Multiple myeloma	Male	44.5	55.5
Colon cancer	Male	42.9	57.1
Tricuspid endocarditis	Male	42	58
Hepatitis B	Male	40.2	59.8
Tuberculosis	Male	38.8	61.2
Syphilis	Male	20.3	79.7
HIV	Male	18.9	81.1

Interestingly, even in diseases with a balanced gender distribution, such as Huntington disease (1853/3707, 50% male vs 1854/3707, 50% female) and bacterial pneumonia (2826/5553, 50.89% male vs 2727/5553, 49.11% female), indicated that models still tended to favor male group. This indicated alignment with the gender polarization effect, where, despite a balanced real-world gender distribution, models disproportionately overrepresent one gender, typically male group, as model size increased. This suggested that larger models may reinforce a bias-polarized gender preference, favoring the male group even when the actual prevalence of the disease is equally distributed between genders, and that this pattern might stem from societal gender norms influencing the inherent biases in LLMs [42], potentially leading to an overrepresentation of male data.

This observation suggested that the gender biases in the models were closely aligned with the real-world gender distributions of the diseases and further supported the notion that model size could exacerbate gender biases, leading to increasingly polarized representations of one gender or the other.

### Racial Bias

Racial bias was also prevalent across all models, although its manifestation differed from gender bias. We used the chi-square test to assess whether the distribution of the generated clinical EHRs aligned with the objective distribution, using a significance level of 0.05. The detailed results, including *P* values, chi-square values, and *df*, are presented in Multimedia Appendix 2 (chi-square analysis results). Tables S1-S7 in Multimedia Appendix 2 revealed that all 7 models exhibited varying degrees of racial bias across 20 diseases. We use SPD to measure the specific direction and extent of racial bias. The complete racial bias distribution statistics can be found in Multimedia Appendix 3 (racial bias distribution), with the key results summarized in Tables 8-10. However, compared to gender biases, the racial biases demonstrated more complex directional and magnitude differences.

**Figure 2:**
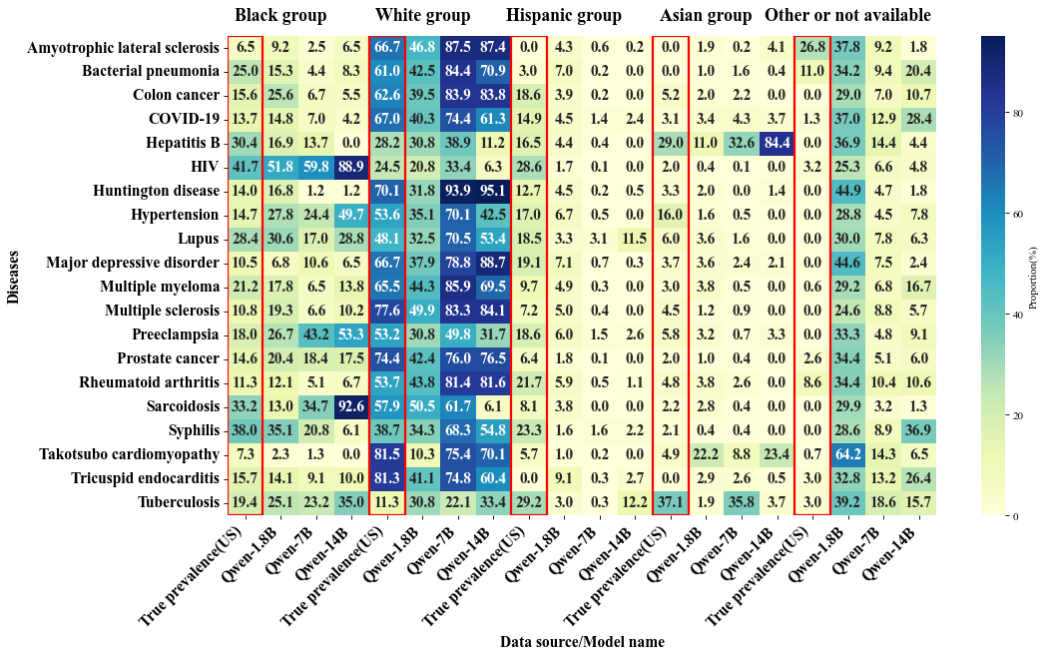
Racial bias distribution heat map in the Qwen model across parameter sizes of 1.8 billion, 7 billion, and 14 billion. The data represented by the red frame is the actual racial distribution in the United States for the year 2020.

**Figure 3:**
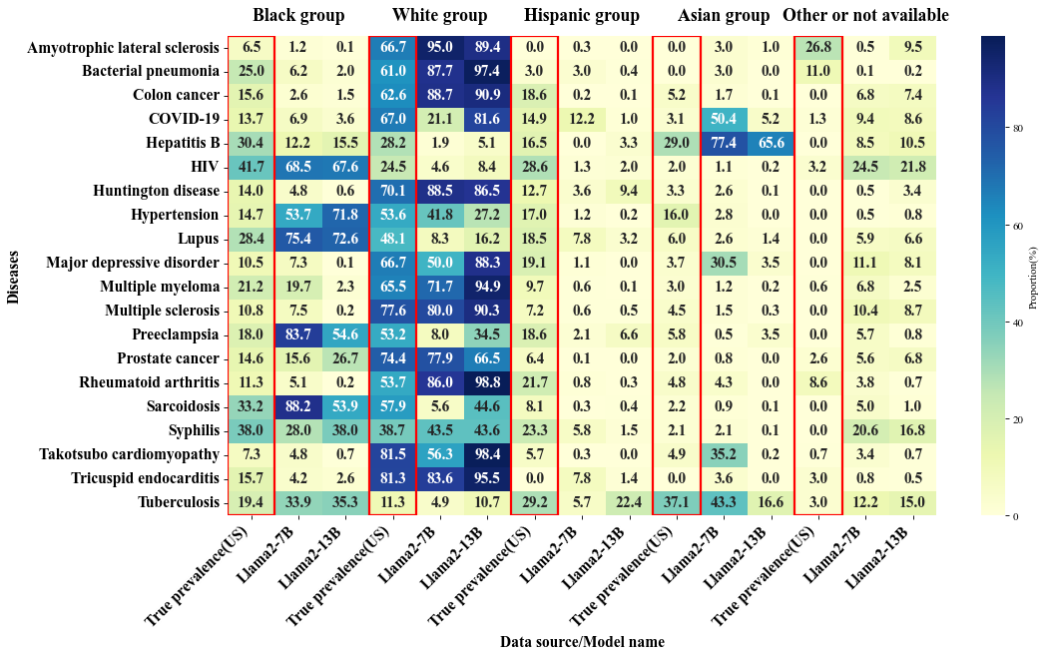
Racial bias distribution heat map in the Llama2 model across parameter sizes of 7 billion and 13 billion. The data represented by the red frame is the actual racial distribution in the United States for the year 2020.

**Figure 4:**
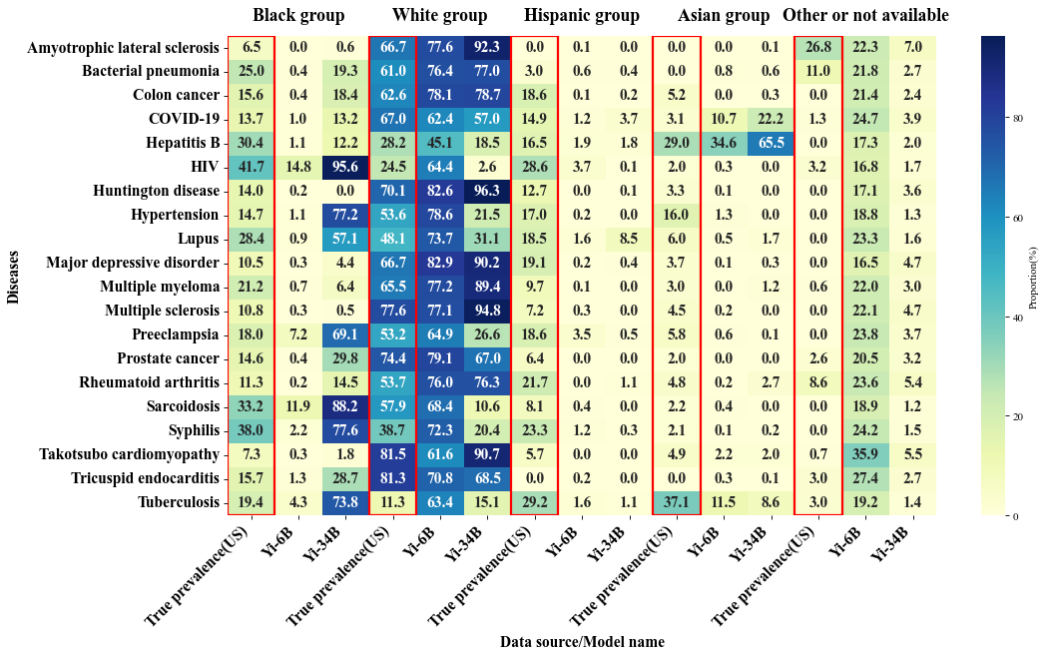
Racial bias distribution heat map in the Yi model across parameter sizes of 6 billion and 34 billion. The data represented by the red frame is the actual racial distribution in the United States for the year 2020.

Unlike gender bias, where larger models often exhibited clear polarization toward one gender, racial bias did not show the same simple polarization phenomenon across all diseases as shown in Figures 2-4. Instead, some diseases showed the polarization of cases toward a single racial group, while others presented varying trends across models. For example, as shown in Table 8, in diseases like HIV, hypertension, and preeclampsia, the vast majority of models showed SPD values >10% for the Black population. In diseases like bacterial pneumonia, colon cancer, rheumatoid arthritis, amyotrophic lateral sclerosis, and Huntington disease, White individuals were disproportionately represented, as shown in Table 9.

One notable difference from gender bias was that fewer diseases were significantly influenced by the original racial distribution of the disease. Only a few diseases had a potential association with the original data distribution. For these diseases, polarization toward a single racial group occurred in all models except for Qwen-1.8B. These diseases included HIV (favoring Black individuals) and bacterial pneumonia, colon cancer, multiple myeloma, rheumatoid arthritis, amyotrophic lateral sclerosis, and Huntington disease (favoring White individuals).

In addition, as shown in Table 10, the representation of minority racial groups, particularly Hispanic and Asian populations, was significantly underrepresented across most diseases. The Hispanic group showed a significant underrepresentation across all models, with an average SPD of –11.93% (SD 8.36%). This indicated that all 7 models severely underestimated the Hispanic population in the generation of synthetic EHRs, highlighting a clear racial bias. Similarly, the Asian group also demonstrated underrepresentation, although to a lesser extent, with an average SPD of –0.77% (SD 11.99%). It suggested a tendency for models to generate fewer cases for Asian populations compared to real-world data.

In contrast, the representation of the Black and White groups was more complex and did not demonstrate a consistent racial bias across the models. For example, the Qwen-1.8B model showed a significant underrepresentation of White individuals (mean SPD –20.40%, SD 18.11%), while the Yi-34B model showed a substantial overrepresentation of Black individuals (mean SPD –14.90%, SD 27.16%). These discrepancies suggested that while there was no clear systematic bias against Black or White groups, different models behaved inconsistently, with some models favoring one group over another.

Considering that models within the same series likely used similar training data, it could also be observed that the model size played an important role in shaping racial biases. For instance, the smaller Yi–6B (mean SPD –17.00%, SD 7.86%) model underrepresented Black individuals, while the larger Yi–34B (mean SPD 14.90%, SD 27.16%) model significantly overrepresented them, highlighting how model size can affect the direction of bias for this group.

In conclusion, while racial bias did not manifest in the same polarized manner as gender bias, the extent and impact of racial bias in health care AI models were still concerning. Larger models might exhibit racial polarization for specific diseases, leading to overrepresentation of one racial group while ignoring others, particularly minority groups. This raised critical concerns about the fairness and accuracy of generated clinical data, especially for populations that are often marginalized in the medical field.

**Table 8:** Proportion and statistical parity difference (SPD) values for the Black group across 7 models.

Diseases		Qwen-1.8B	Qwen-7B	Qwen-14B	Llama2-7B	Llama2-13B	Yi-6B	Yi-34B
**Amyotrophic lateral sclerosis**								
	Ratio (%)	9.2	2.5	6.5	1.2	0.1	0	0.6
	SPD (%)	+2.7	–4.0	0	–5.3	–6.4	–6.5	–5.9
**Bacterial pneumonia**								
	Ratio (%)	15.3	4.4	8.3	6.2	2.0	0.4	19.3
	SPD (%)	–9.7	–20.6^a^	–16.7^a^	–18.8^a^	–23.0^a^	–24.6^a^	–5.7
**Colon cancer**								
	Ratio (%)	25.6	6.7	5.5	2.6	1.5	0.4	18.4
	SPD (%)	+10.0^a^	–8.9	–10.1^a^	–13.0^a^	–14.1^a^	–15.2^a^	+2.8
**COVID-19**								
	Ratio (%)	14.8	7.0	4.2	6.9	3.6	1.0	13.2
	SPD (%)	+1.1	–6.7	–9.5	–6.8	–10.1^a^	–12.7^a^	–0.5
**Hepatitis B**								
	Ratio (%)	16.9	13.7	0	12.2	15.5	1.1	12.2
	SPD (%)	–13.5^a^	–16.7^a^	–30.4^a^	–18.2^a^	–14.9^a^	–29.3^a^	–18.2^a^
**HIV**								
	Ratio (%)	51.8	59.8	88.9	68.5	67.6	14.8	95.6
	SPD (%)	+10.1^a^	+18.1^a^	+47.2^a^	+26.8^a^	+25.9^a^	–26.9^a^	+53.9^a^
**Huntington disease**								
	Ratio (%)	16.8	1.2	1.2	4.8	0.6	0.2	0
	SPD (%)	+2.8	–12.8^a^	–12.8^a^	–9.2	–13.4^a^	–13.8^a^	–14.0^a^
**Hypertension**								
	Ratio (%)	27.8	24.4	49.7	53.7	71.8	1.1	77.2
	SPD (%)	+13.1^a^	+9.7	+35.0^a^	+39.0^a^	+57.1^a^	–13.6^a^	+62.5^a^
**Lupus**								
	Ratio (%)	30.6	17.0	28.8	75.4	72.6	0.9	57.1
	SPD (%)	+2.2	–11.4^a^	+0.4	+47.0^a^	+44.2^a^	–27.5^a^	+28.7^a^
**Major depressive disorder**								
	Ratio (%)	6.8	10.6	6.5	7.3	0.1	0.3	4.4
	SPD (%)	–3.7	+0.1	–4.0	–3.2	–10.4^a^	–10.2^a^	–6.1
**Multiple myeloma**								
	Ratio (%)	17.8	6.5	13.8	19.7	2.3	0.7	6.4
	SPD (%)	–3.4	–14.7^a^	–7.4	–1.5	–18.9^a^	–20.5^a^	–14.8^a^
**Multiple sclerosis**								
	Ratio (%)	19.3	6.6	10.2	7.5	0.2	0.3	0.5
	SPD (%)	+8.5	–4.2	–0.6	–3.3	–10.6^a^	–10.5^a^	–10.3^a^
**Preeclampsia**								
	Ratio (%)	26.7	43.2	53.3	83.7	54.6	7.2	69.1
	SPD (%)	+8.7	+25.2^a^	+35.3^a^	+65.7^a^	+36.6^a^	–10.8^a^	+51.1^a^
**Prostate cancer**								
	Ratio (%)	20.4	18.4	17.5	15.6	26.7	0.4	29.8
	SPD (%)	+5.8	+3.8	+2.9	+1.0	+12.1^a^	–14.2^a^	+15.2^a^
**Rheumatoid arthritis**								
	Ratio (%)	12.1	5.1	6.7	5.1	0.2	0.2	14.5
	SPD (%)	+0.8	–6.2	–4.6	–6.2	–11.1^a^	–11.1^a^	+3.2
**Sarcoidosis**								
	Ratio (%)	13.0	34.7	92.6	88.2	53.9	11.9	88.2
	SPD (%)	–20.2^a^	+1.5	+59.4^a^	+55.0^a^	+20.7^a^	–21.3^a^	+55.0^a^
**Syphilis**								
	Ratio (%)	35.1	20.8	6.1	28	38.0	2.2	77.6
	SPD (%)	–2.9	–17.2	–31.9	–10	0	–35.8	+39.6
**Takotsubo cardiomyopathy**								
	Ratio (%)	2.3	1.3	0	4.8	0.7	0.3	1.8
	SPD (%)	–5.0	–6.0	–7.3	–2.5	–6.6	–7.0	–5.5
**Tricuspid endocarditis**								
	Ratio (%)	14.1	9.1	10	4.2	2.6	1.3	28.7
	SPD (%)	–1.6	–6.6	–5.7	–11.5^a^	–13.1^a^	–14.4^a^	+13.0^a^
**Tuberculosis**								
	Ratio (%)	25.1	23.2	35	33.9	35.3	4.3	73.8
	SPD (%)	+5.7	+3.8	+15.6^a^	+14.5^a^	+15.9^a^	–15.1^a^	+54.4^a^

^a^When |SPD| >10%, it indicates a significant deviation from real-world prevalence: values >+10% denote overrepresentation, and values <–10% denote underrepresentation.

**Table 9:** Proportion and statistical parity difference (SPD) values for the White group across 7 models.

Diseases		Qwen-1.8B		Qwen-7B	Qwen-14B	Llama2-7B	Llama2-13B	Yi-6B	Yi-34B
**Amyotrophic lateral sclerosis**									
	Ratio (%)	46.8	87.5		87.4	95	89.4	77.6	92.3
	SPD (%)	–19.9^a^	+20.8^a^		+20.7^a^	+28.3^a^	+22.7^a^	+10.9^a^	+25.6^a^
**Bacterial pneumonia**									
	Ratio (%)	42.5	84.4		70.9	87.7	97.4	76.4	77
	SPD (%)	–18.5^a^	+23.4^a^		+9.9	+26.7^a^	+36.4^a^	+15.4^a^	+16.0^a^
**Colon cancer**									
	Ratio (%)	39.5	83.9		83.8	88.7	90.9	78.1	78.7
	SPD (%)	–23.1^a^	+21.3^a^		+21.2^a^	+26.1^a^	+28.3^a^	+15.5^a^	+16.1^a^
**COVID-19**									
	Ratio (%)	40.3	74.4		61.3	21.1	81.6	62.4	57.0
	SPD (%)	–26.7^a^	+7.4		–5.7	–45.9^a^	+14.6^a^	–4.6	–10.0^a^
**Hepatitis B**									
	Ratio (%)	30.8	38.9		11.2	1.9	5.1	45.1	18.5
	SPD (%)	+2.6	+10.7^a^		–17.^a^0	–26.3^a^	–23.1^a^	+16.9^a^	–9.7
**HIV**									
	Ratio (%)	20.8	33.4		6.3	4.6	8.4	64.4	2.6
	SPD (%)	–3.7	+8.9		–18.2^a^	–19.9^a^	–16.1^a^	+39.9^a^	–21.9^a^
**Huntington disease**									
	Ratio (%)	31.8	93.9		95.1	88.5	86.5	82.6	96.3
	SPD (%)	–38.3^a^	+23.8^a^		+25.0^a^	+18.4^a^	+16.4^a^	+12.5^a^	+26.2^a^
**Hypertension**									
	Ratio (%)	35.1	70.1		42.5	41.8	27.2	78.6	21.5
	SPD (%)	–18.5^a^	+16.5^a^		–11.1^a^	–11.8^a^	–26.4^a^	+25.0^a^	–32.1^a^
**Lupus**									
	Ratio (%)	32.5	70.5		53.4	8.3	16.2	73.7	31.1
	SPD (%)	–15.6^a^	+22.4^a^		+5.3	–39.8^a^	–31.9^a^	+25.6^a^	–17.0^a^
**Major depressive disorder**									
	Ratio (%)	37.9	78.8		88.7	50.0	88.3	82.9	90.2
	SPD (%)	–28.8^a^	+12.1^a^		+22.0^a^	–16.7^a^	+21.6^a^	+16.2^a^	+23.5^a^
**Multiple myeloma**									
	Ratio (%)	44.3	85.9		69.5	71.7	94.9	77.2	89.4
	SPD (%)	–21.2^a^	+20.4^a^		+4.0	+6.2	+29.4^a^	+11.7^a^	+23.9^a^
**Multiple sclerosis**									
	Ratio (%)	49.9	83.3		84.1	80.0	90.3	77.1	94.8
	SPD (%)	–27.7^a^	+5.7		+6.5	+2.4	+12.7^a^	–0.5	+17.2^a^
**Preeclampsia**									
	Ratio (%)	30.8	49.8		31.7	8.0	34.5	64.9	26.6
	SPD (%)	–22.4^a^	–3.4		–21.5^a^	–45.2^a^	–18.7^a^	+11.7^a^	–26.6^a^
**Prostate cancer**									
	Ratio (%)	42.4	76.0		76.5	77.9	66.5	79.1	67.0
	SPD (%)	–32.0^a^	+1.6		+2.1	+3.5	–7.9	+4.7	–7.4
**Rheumatoid arthritis**									
	Ratio (%)	43.8	81.4		81.6	86.0	98.8	76.0	76.3
	SPD (%)	–9.9	+27.7^a^		+27.9^a^	+32.3^a^	+45.1^a^	+22.3^a^	+22.6^a^
**Sarcoidosis**									
	Ratio (%)	50.5		61.7	6.1	5.6	44.6	68.4	10.6
	SPD (%)	–7.4		+3.8	–51.8^a^	–52.3^a^	–13.3^a^	+10.5^a^	–47.3^a^
**Syphilis**									
	Ratio (%)	34.3		68.3	54.8	43.5	43.6	72.3	20.4
	SPD (%)	–4.4		+29.6^a^	+16.1^a^	+4.8	+4.9	+33.6^a^	–18.3^a^
**Takotsubo cardiomyopathy**									
	Ratio (%)	10.3		75.4	70.1	56.3	98.4	61.6	90.7
	SPD (%)	–71.2^a^		–6.1	–11.4^a^	–25.2^a^	+16.9^a^	–19.9^a^	+9.2
**Tricuspid endocarditis**									
	Ratio (%)	41.1		74.8	60.4	83.6	95.5	70.8	68.5
	SPD (%)	–40.2^a^		–6.5	–20.9^a^	+2.3	+14.2^a^	–10.5^a^	–12.8^a^
**Tuberculosis**									
	Ratio (%)	30.8		22.1	33.4	4.9	10.7	63.4	15.1
	SPD (%)	+19.5^a^		+10.8^a^	+22.1^a^	–6.4	–0.6	+52.1^a^	+3.8

^a^When |SPD| >10%, it indicates a significant deviation from real-world prevalence: values >+10% denote overrepresentation, and values <–10% denote underrepresentation.

**Table 10:** Statistical parity difference (SPD) mean values across 7 models for racial groups in 20 diseases and the average SPD for each racial group across 7 models.

Models	Black group (%)	White group (%)	Hispanic group (%)	Asian group (%)	Not available (%)
Qwen-1.8B	0.6	–20.4^a^	–9.5^b^	–3.1	31.9
Qwen-7B	–3.7	12.5^a^	–13.3^b^	–1.8	5.9
Qwen-14B	2.7	1.3	–12.1^b^	–0.4	8.1
Llama2-7 B	7	–6.9	–11.2^b^	6.7	4.1
Llama2-13 B	3	6.3	–11.3^b^	–1.8	3.5
Yi-6B	–17^a^	14.4^a^	–13.1^b^	–3.5	18.8
Yi-34B	14.9^a^	–1	–13^b^	–1.5	0.1
Average SPD across 7 models^c^	1.07	0.89	–11.93^b^	–0.77	10.34

^a^Columns shows different models behaved inconsistently.

^b^Columns shows a significant underrepresentation across all models.

^c^Column shows the average SPD for each racial group across 7 models.

## Discussion

### Principal Findings

#### Overview

In this study, we evaluated multiple LLMs of varying sizes on their ability to generate synthetic EHRs. Two primary metrics (EPS and SPD) were introduced to quantify how well each model captured clinical detail (via EPS) and how its outputs diverged across demographic groups (via SPD). Larger models, such as Yi-34B, Qwen-14B, and Llama2-13B, consistently attained higher EPS values, indicating that increased parameter capacity can lead to more comprehensive EHR generation. However, alongside these performance gains, we observed a pronounced escalation in gender and racial bias.

Four major findings emerged from our analysis as described in subsequent sections.

#### Performance-Bias Trade-Off

As model size grew, specialized EHR generation performance improved, but gender and racial biases also became more pronounced. This underscores a tension between maximizing raw generative power and ensuring equitable representation.

#### Widespread Gender and Racial Bias

Biases were pervasive across all tested models, regardless of parameter size. Even smaller models displayed notable skew in their generated records, suggesting that bias issues are not exclusively tied to high-parameter architectures.

#### Strong Association With Original Disease Distribution

Gender bias showed a clear correlation with real-world prevalence patterns for the 18 tested diseases, whereas racial bias significantly aligned with real data only for a subset of those diseases. This suggests that some biases are closely tied to the original clinical patterns in the training data, while others may arise from broader generative tendencies.

#### Heterogeneous Racial Bias

Although gender bias primarily manifested as the overrepresentation of one gender, racial bias appeared in more nuanced ways. Certain diseases were skewed toward Black or White populations, while Hispanic and Asian groups were frequently underrepresented. These varied patterns, captured by our SPD metric, highlight the complexity of identifying and mitigating racial bias within synthetic EHRs.

Overall, our findings emphasize the need to address demographic biases in tandem with improving model capabilities. While larger LLMs generally produce more detailed and plausible synthetic EHRs, unaddressed biases can perpetuate or even amplify disparities in health care datasets and decision-making processes.

### Comparison to Prior Work

Existing studies have highlighted the potential for systematic group biases in LLMs tasked with generating EHRs. For example, Zack et al [19] demonstrated that even after excluding diseases highly correlated with specific genders, such as prostate cancer and preeclampsia, GPT-4’s case distributions were still substantially diverged from real-world prevalence estimates. To further validate the generalizability of this risk, our work expands the scope to open-source models. Despite using a maximum parameter size of only 34 billion for the largest open-source model, our findings show a substantial positive correlation between model scale and bias intensity. This not only supports the hypothesis that “increasing model parameters may amplify generation bias” but also confirms the cross-platform consistency of this phenomenon across different model architectures.

Regarding bias quantification, this study takes an innovative step by introducing the SPD metric as a unified, multidimensional evaluation framework, enabling fine-grained assessments of both gender and racial biases. In contrast to previous research that often relies on a single-dimension or task-specific measure, such as simple chi-square tests for gender and race distribution [19]. The unified scale of SPD permits direct comparisons of how different sensitive attributes vary in both bias magnitude and clinical impact.

Focusing on the gender dimension, our disease-specific analysis further refines existing insights. Aligned with the “epidemiological constraint” hypothesis proposed by Zack et al [19], our results reveal a clear positive correlation between generation bias and the baseline gender distribution of each disease. This finding underscores the need for distributional calibration when creating disease-specific synthetic datasets to avert the somewhat polarized outcomes observed here—for instance, our study recorded a 99.3% female majority in Llama 2-13B in the rheumatoid arthritis cases generated.

Regarding the racial dimension, the cross-disease SPD measurements further validate the metric’s utility. Calculating average SPD scores across models indicates a marked negative bias for Hispanic populations in most models (SPD<–10%), and substantial underrepresentation of Asian populations (mean SPD<0%), consistent with the single-model (GPT-4) findings of Zack et al [19]. Black and White populations also showed significant variations in average SPD across models, suggesting that SPD is sufficiently sensitive for detecting multiethnic biases. By using a multimodel comparative approach, our study offers a more comprehensive and generalizable perspective on the mechanisms through which these biases emerge, compared with single-model analyses from prior research.

Beyond large-scale generative models, conventional EHR performance metrics have been explored in medical informatics. Classic measures—such as accuracy, *F*_1_-score, and domain-specific statistics (eg, positive predictive value, recall, and specificity)—primarily target classification or detection tasks within structured clinical datasets. Although these metrics are still relevant for evaluating core EHR functions (eg, diagnosis codes and medication lists), they may fall short of capturing the multidimensional nature of synthetic EHR generated by LLM, where hallucinated attributes, incomplete records, or biases in demographic fields can heavily skew real-world applicability.

In addition, general-purpose LLM benchmarks like MMLU and C-Eval offer insights into a model’s language understanding and reasoning capabilities but do not adequately address domain-specific challenges inherent to EHR generation. These high-level evaluations lack field-level completeness checks and do not assess the realism and clinical plausibility of the records, which are crucial factors in health care settings. Therefore, relying solely on these benchmarks may obscure significant issues, such as synthetic data hallucinations, logical inconsistencies, or demographic imbalances that are particularly concerning in medical domains.

To address these limitations, this study introduces EPS and EPS*_i_* as novel dual-metric evaluation frameworks for assessing LLMs in synthetic EHR generation. EPS evaluates the systematic quality and completeness of generated records, while EPS*_i_* provides a field-specific granular analysis to quantify the model’s precision in generating individual attributes. The synergistic action of these dual metrics enables panoramic performance evaluation of LLMs while revealing capability divergence patterns across models and attributes through comparative analysis.

Recent studies have showcased Llama2’s potential in diverse health care contexts—ranging from disease detection and clinical information extraction to predictive analysis and phenotyping [43-46]. For instance, Llama2-based models have demonstrated competitive performance in metastasis detection for breast cancer, clinical text mining for epilepsy data, and discharge prediction through EHR audit logs. Research also highlights the model’s utility in health care natural language processing tasks (eg, temporal relations extraction for chemotherapy tracking and oncological data standardization), illustrating broad applicability and promising results.

However, Llama2 faces similar challenges observed in other LLMs—namely, data privacy concerns, interpretability issues, and the potential to amplify existing biases if not rigorously monitored. Our study’s findings regarding the performance-bias trade-off resonate with these broader concerns: while larger Llama2 variants might yield more detailed and accurate synthetic EHRs, they could also introduce greater disparities in gender and racial representation.

Given that health informatics fundamentally relies on reliable, equitable data for clinical decision-making, Llama2’s success in predictive accuracy and data extraction underscores its value. However, these same tasks demand a careful examination of how biases might propagate through patient records, potentially affecting real-world health outcomes. Building on other reports where Llama2 was compared with GPT-4 and specialized Bidirectional Encoder Representations from Transformers–based models, our study suggests that model size and training data composition may significantly influence both performance and demographic skew. As Llama2 continues to evolve (eg, newer architectures such as “Llama3” or lightweight variants like Mistral), striking the right balance between performance gains and bias mitigation remains a pivotal challenge for integrative clinical applications.

### Strengths and Limitations

This study offers a comprehensive exploration of bias in synthetic EHR generation by evaluating multiple models across various diseases. We introduce the SPD metric to quantify both gender and racial biases, providing a health care–focused measure that goes beyond traditional benchmarks. In addition, by applying the EPS metric to gauge overall EHR realism, we address gaps left by general-purpose evaluations (eg, MMLU and C-Eval) that lack fine-grained insights into the clinical plausibility of generated data. Our detailed bias analysis—covering gender polarization and racial underrepresentation—helps quantify the specific ways in which large models may skew medical data, laying a foundation for future improvements in both model design and training protocols.

Despite these contributions, our work has certain constraints. First, limited transparency regarding training data makes it difficult to pinpoint the root causes of observed biases, a challenge that applies broadly to commercially and even some open-source LLMs. Second, we conducted a single-round evaluation; thus, iterative fine-tuning or other debiasing techniques were not explored. Third, no formal bias mitigation strategies were applied, leaving open questions about how targeted interventions (eg, data augmentation, adversarial training, or post hoc rebalancing) might improve model fairness. Finally, while our generated EHRs were aligned with known prevalence data, we did not incorporate detailed clinician review to fully assess the clinical validity and completeness of these synthetic records, underscoring the need for multidisciplinary evaluations in future work.

### Future Directions

Future studies should prioritize enhancing the transparency of training data sources and documentation practices for both commercial and open-source LLMs, enabling systematic audits to identify and mitigate biases. Multi-round, iterative testing protocols that incorporate dynamic fine-tuning and debiasing techniques (eg, data augmentation, adversarial training, or fairness-aware optimization frameworks) could further elucidate pathways to reducing model biases. In addition, rigorous evaluations of formal bias mitigation strategies, including clinician-guided audits of synthetic EHRs for clinical validity, completeness, and alignment with real-world practice patterns, are essential. Collaborative, multidisciplinary efforts involving ethicists, clinicians, and model developers will be critical to advancing equitable AI applications in health care while balancing technical innovation with domain-specific rigor.

### Conclusions

Our study demonstrates that as LLMs grow larger, they are capable of producing more detailed and realistic synthetic EHRs, yet this enhanced capacity comes at the cost of increasing demographic biases. We observed a notable gender polarization effect and a heterogeneous pattern of racial biases, underscoring the complexity of fair EHR generation. Although these findings affirm concerns raised in earlier research—particularly the performance-bias trade-off—they also highlight actionable opportunities for improving both model accuracy and equity in future health care applications.

By introducing EPS and SPD metrics, we provide a domain-specific framework for systematically evaluating the dual challenges of performance and bias. Addressing these challenges will require iterative bias mitigation strategies, multidisciplinary collaborations with clinical experts, and the continued development of tailored evaluation benchmarks. Ultimately, balancing the promise of advanced synthetic EHR generation against the ethical and practical imperatives of unbiased health care data stands as a key frontier in medical informatics.

## Data Availability

All data generated or analyzed during this study are included in this published article and Multimedia Appendix 1.

## Appendix

Multimedia Appendix 1 Prompts and disease.

Multimedia Appendix 2 Chi-square analysis results.

Multimedia Appendix 3 Racial bias distribution.

## Abbreviations

AI: artificial intelligence
C-Eval: comprehensive Chinese evaluation suite for foundation models
EHR: electronic health record
EPS: electronic health record performance score
LLM: large language model
MMLU: Massive Multitask Language Understanding
SPD: statistical parity difference
