# Supplementary material for: Evaluation and Bias Analysis of Large Language Models in Generating Synthetic Electronic Health Records: Comparative Study
Source: J Med Internet Res. 2025 May 12;27:e65317. doi: 10.2196/65317 (PMC12107208; doi:10.2196/65317)
Supplement: Multimedia Appendix 2 [file jmir_v27i1e65317_app2.docx]

## Multimedia Appendix 2: Chi-Square Analysis Results

**Table 1.** Chi-square analysis of gender and race distribution differences between Llama2-7B generated clinical EHRs and real-world data across 20 diseases (1000 cases per disease, N=20,000)

| **Diseases** | ${df}_{race}$ | $P_{race}$ | ${\chi^{2}}_{race}$ | ${df}_{gender}$ | $P_{gender}$ | ${\chi^{2}}_{gender}$ |
| --- | --- | --- | --- | --- | --- | --- |
| Amyotrophic Lateral Sclerosis | 2^b^ | <.001 | 329.591 | 1 | <.001 | 349.132 |
| Bacterial Pneumonia | 3^c^ | <.001 | 261.468 | 1 | <.001 | 282.241 |
| Colon cancer | 3^d^ | <.001 | 313.784 | 1 | <.001 | 279.615 |
| COVID-19 | 4^e^ | <.001 | 743.773 | 1 | <.001 | 368.270 |
| Hepatitis B | 3^f^ | <.001 | 649.638 | 1 | <.001 | 245.250 |
| HIV | 4^g^ | <.001 | 616.123 | 1 | <.001 | 18.1674 |
| Huntington Disease | 3^h^ | <.001 | 117.572 | 1 | <.001 | 183.620 |
| Hypertension | 3^i^ | <.001 | 464.976 | 1 | <.001 | 248.604 |
| Lupus | 3^j^ | <.001 | 530.497 | 1 | <.001 | 78.903 |
| Major Depressive Disorder | 3^k^ | <.001 | 376.097 | 1 | <.001 | 32.807 |
| Multiple Myeloma | 4^l^ | <.001 | 143.676 | 1 | <.001 | 368.093 |
| Multiple Sclerosis | 4^m^ | <.001 | 180.680 | 1 | <.001 | 73.769 |
| Preeclampsia | 3^n^ | <.001 | 906.027 | N/A^a^ | N/A^a^ | N/A^a^ |
| Prostate cancer | 4^o^ | <.001 | 77.979 | N/A^a^ | N/A^a^ | N/A^a^ |
| Rheumatoid Arthritis | 4^p^ | <.001 | 310.020 | 1 | <.001 | 41.358 |
| Scaecoidosis | 3^q^ | <.001 | 730.705 | 1 | <.001 | 125.800 |
| Syphilis | 3^r^ | <.001 | 81.6594 | 1 | <.001 | 129.839 |
| Takotsubo cardiomyopathy | 4^s^ | <.001 | 346.857 | 1 | <.001 | 48.153 |
| Tricuspid Endocarditis | 2^t^ | <.001 | 66.4703 | 1 | <.001 | 480.968 |
| Tuberculosis | 4^u^ | <.001 | 282.952 | 1 | <.001 | 201.962 |

^a^There are N/A values where we are unable to assess statistical significance when the conditions are present in only one gender.

^b^Data were obtained directly from the source. Real data for two variables(Asian and Hispanic) are missing or unavailable, as referenced in [1].

^c^Data were obtained directly from the source. Real data for one variables(Asian) is missing or unavailable, as referenced in [2].

^d^Data were obtained calculated from relevant data within the source. Real data for one variables (Others) is missing or unavailable, as referenced in [3].

^e^Data were obtained directly from the source. No variables were missing or unavailable, as referenced in [4].

^f^Data were obtained calculated from relevant data within the source. Real data for one variables (Others) is missing or unavailable, as referenced in [5].

^g^Data were obtained calculated from relevant data within the source. No variables were missing or unavailable, as referenced in [6].

^h^Data were obtained directly from the source. Real data for one variable (Others) is missing or unavailable, as referenced in [7].

^i^Data were obtained calculated from relevant data within the source. Real data for one variable (Others) is missing or unavailable, as referenced in [8].

^j^Data were obtained calculated from relevant data within the source. Real data for one variables (Others) is missing or unavailable, as referenced in [9].

^k^Data were obtained directly from the source. Real data for one variable(Others) is missing or unavailable, as referenced in [10].

^l^Data were obtained calculated from relevant data within the source. No variables were missing or unavailable, as referenced in [11].

^m^Data were obtained calculated from relevant data within the source. No variables were missing or unavailable, as referenced in [12].

^n^Data were obtained calculated from relevant data within the source. Real data for one variable (Others) is missing or unavailable, as referenced in [13].

^o^Data were obtained calculated from relevant data within the source. No variables were missing or unavailable, as referenced in [14].

^p^Data were obtained calculated from relevant data within the source. No variables were missing or unavailable, as referenced in [15].

^q^Data were obtained calculated from relevant data within the source. Real data for one variable (Others) is missing or unavailable, as referenced in [16].

^r^Data were obtained calculated from relevant data within the source. Real data for one variable (Others) is missing or unavailable, as referenced in [17].

^s^Data were obtained calculated from relevant data within the source. No variables were missing or unavailable, as referenced in [18].

^t^Data were obtained directly from the source. Real data for two variables (Asian and Hispanic) are missing or unavailable, as referenced in [19].

^u^Data were obtained calculated from relevant data within the source. No variables were missing or unavailable, as referenced in [20].

**Table 2.** Chi-square analysis of gender and race distribution differences between Llama2-13B generated clinical EHRs and real-world data across 20 diseases (1000 cases per disease, N=20,000)

| **Diseases** | ${df}_{race}$ | $P_{race}$ | ${\chi^{2}}_{race}$ | ${df}_{gender}$ | $P_{gender}$ | ${\chi^{2}}_{gender}$ |
| --- | --- | --- | --- | --- | --- | --- |
| Amyotrophic Lateral Sclerosis | 2^b^ | <.001 | 176.429 | 1 | <.001 | 517.908 |
| Bacterial Pneumonia | 3^c^ | <.001 | 403.598 | 1 | <.001 | 472.470 |
| Colon cancer | 3^d^ | <.001 | 371.850 | 1 | <.001 | 454.066 |
| COVID-19 | 4^e^ | <.001 | 254.197 | 1 | <.001 | 392.784 |
| Hepatitis B | 3^f^ | <.001 | 403.595 | 1 | <.001 | 389.245 |
| HIV | 4^g^ | <.001 | 523.736 | 1 | <.001 | 126.190 |
| Huntington Disease | 3^h^ | <.001 | 169.378 | 1 | <.001 | 424.010 |
| Hypertension | 3^i^ | <.001 | 783.293 | 1 | <.001 | 491.380 |
| Lupus | 3^j^ | <.001 | 468.421 | 1 | <.001 | 98.236 |
| Major Depressive Disorder | 3^k^ | <.001 | 296.740 | 1 | <.001 | 198.804 |
| Multiple Myeloma | 4^l^ | <.001 | 336.789 | 1 | <.001 | 534.687 |
| Multiple Sclerosis | 4^m^ | <.001 | 293.148 | 1 | <.001 | 222.556 |
| Preeclampsia | 3^n^ | <.001 | 286.643 | N/A^a^ | N/A^a^ | N/A^a^ |
| Prostate cancer | 4^o^ | <.001 | 142.117 | N/A^a^ | N/A^a^ | N/A^a^ |
| Rheumatoid Arthritis | 4^p^ | <.001 | 562.055 | 1 | <.001 | 261.745 |
| Scaecoidosis | 3^q^ | <.001 | 154.199 | 1 | <.001 | 287.338 |
| Syphilis | 3^r^ | <.001 | 166.641 | 1 | <.001 | 158.390 |
| Takotsubo cardiomyopathy | 4^s^ | <.001 | 170.435 | 1 | <.001 | 80.009 |
| Tricuspid Endocarditis | 2^t^ | <.001 | 121.541 | 1 | <.001 | 252.419 |
| Tuberculosis | 4^u^ | <.001 | 212.983 | 1 | <.001 | 411.107 |

^a^There are N/A values where we are unable to assess statistical significance when the conditions are present in only one gender.

^b^Data were obtained directly from the source. Real data for two variables(Asian and Hispanic) are missing or unavailable, as referenced in [1].

^c^Data were obtained directly from the source. Real data for one variables(Asian) is missing or unavailable, as referenced in [2].

^d^Data were obtained calculated from relevant data within the source. Real data for one variables (Others) is missing or unavailable, as referenced in [3].

^e^Data were obtained directly from the source. No variables were missing or unavailable, as referenced in [4].

^f^Data were obtained calculated from relevant data within the source. Real data for one variables (Others) is missing or unavailable, as referenced in [5].

^g^Data were obtained calculated from relevant data within the source. No variables were missing or unavailable, as referenced in [6].

^h^Data were obtained directly from the source. Real data for one variable (Others) is missing or unavailable, as referenced in [7].

^i^Data were obtained calculated from relevant data within the source. Real data for one variable (Others) is missing or unavailable, as referenced in [8].

^j^Data were obtained calculated from relevant data within the source. Real data for one variables (Others) is missing or unavailable, as referenced in [9].

^k^Data were obtained directly from the source. Real data for one variable(Others) is missing or unavailable, as referenced in [10].

^l^Data were obtained calculated from relevant data within the source. No variables were missing or unavailable, as referenced in [11].

^m^Data were obtained calculated from relevant data within the source. No variables were missing or unavailable, as referenced in [12].

^n^Data were obtained calculated from relevant data within the source. Real data for one variable (Others) is missing or unavailable, as referenced in [13].

^o^Data were obtained calculated from relevant data within the source. No variables were missing or unavailable, as referenced in [14].

^p^Data were obtained calculated from relevant data within the source. No variables were missing or unavailable, as referenced in [15].

^q^Data were obtained calculated from relevant data within the source. Real data for one variable (Others) is missing or unavailable, as referenced in [16].

^r^Data were obtained calculated from relevant data within the source. Real data for one variable (Others) is missing or unavailable, as referenced in [17].

^s^Data were obtained calculated from relevant data within the source. No variables were missing or unavailable, as referenced in [18].

^t^Data were obtained directly from the source. Real data for two variables (Asian and Hispanic) are missing or unavailable, as referenced in [19].

^u^Data were obtained calculated from relevant data within the source. No variables were missing or unavailable, as referenced in [20].

**Table 3.** Chi-square analysis of gender and race distribution differences between Qwen-1.8B generated clinical EHRs and real-world data across 20 diseases (1000 cases per disease, N=20,000)

| **Diseases** | ${df}_{race}$ | $P_{race}$ | ${\chi^{2}}_{race}$ | ${df}_{gender}$ | $P_{gender}$ | ${\chi^{2}}_{gender}$ |
| --- | --- | --- | --- | --- | --- | --- |
| Amyotrophic Lateral Sclerosis | 2^b^ | <.001 | 54.385 | 1 | <.001 | 89.328 |
| Bacterial Pneumonia | 3^c^ | <.001 | 190.778 | 1 | <.001 | 52.410 |
| Colon cancer | 3^d^ | <.001 | 110.847 | 1 | .89 | 0.018 |
| COVID-19 | 4^e^ | <.001 | 456.475 | 1 | <.001 | 28.918 |
| Hepatitis B | 3^f^ | <.001 | 70.971 | 1 | <.001 | 50.436 |
| HIV | 4^g^ | <.001 | 433.681 | 1 | <.001 | 105.138 |
| Huntington Disease | 3^h^ | <.001 | 45.022 | 1 | <.001 | 65.765 |
| Hypertension | 3^i^ | <.001 | 158.119 | 1 | .75 | 0.100 |
| Lupus | 3^j^ | <.001 | 68.571 | 1 | .22 | 1.485 |
| Major Depressive Disorder | 3^k^ | .02 | 10.262 | 1 | <.001 | 21.718 |
| Multiple Myeloma | 4^l^ | <.001 | 335.052 | 1 | <.001 | 50.413 |
| Multiple Sclerosis | 4^m^ | <.001 | 352.988 | 1 | .007 | 7.214 |
| Preeclampsia | 3^n^ | <.001 | 80.435 | N/A^a^ | N/A^a^ | N/A^a^ |
| Prostate cancer | 4^o^ | <.001 | 399.161 | N/A^a^ | N/A^a^ | N/A^a^ |
| Rheumatoid Arthritis | 4^p^ | <.001 | 256.525 | 1 | .01 | 5.981 |
| Scaecoidosis | 3^q^ | <.001 | 42.527 | 1 | .3 | 1.174 |
| Syphilis | 3^r^ | <.001 | 115.438 | 1 | <.001 | 78.080 |
| Takotsubo cardiomyopathy | 4^s^ | <.001 | 1343.548 | 1 | <.001 | 53.531 |
| Tricuspid Endocarditis | 2^t^ | <.001 | 364.401 | 1 | <.001 | 52.399 |
| Tuberculosis | 4^u^ | <.001 | 937.456 | 1 | <.001 | 58.926 |

^a^There are N/A values where we are unable to assess statistical significance when the conditions are present in only one gender.

^b^Data were obtained directly from the source. Real data for two variables(Asian and Hispanic) are missing or unavailable, as referenced in [1].

^c^Data were obtained directly from the source. Real data for one variables(Asian) is missing or unavailable, as referenced in [2].

^d^Data were obtained calculated from relevant data within the source. Real data for one variables (Others) is missing or unavailable, as referenced in [3].

^e^Data were obtained directly from the source. No variables were missing or unavailable, as referenced in [4].

^f^Data were obtained calculated from relevant data within the source. Real data for one variables (Others) is missing or unavailable, as referenced in [5].

^g^Data were obtained calculated from relevant data within the source. No variables were missing or unavailable, as referenced in [6].

^h^Data were obtained directly from the source. Real data for one variable (Others) is missing or unavailable, as referenced in [7].

^i^Data were obtained calculated from relevant data within the source. Real data for one variable (Others) is missing or unavailable, as referenced in [8].

^j^Data were obtained calculated from relevant data within the source. Real data for one variables (Others) is missing or unavailable, as referenced in [9].

^k^Data were obtained directly from the source. Real data for one variable(Others) is missing or unavailable, as referenced in [10].

^l^Data were obtained calculated from relevant data within the source. No variables were missing or unavailable, as referenced in [11].

^m^Data were obtained calculated from relevant data within the source. No variables were missing or unavailable, as referenced in [12].

^n^Data were obtained calculated from relevant data within the source. Real data for one variable (Others) is missing or unavailable, as referenced in [13].

^o^Data were obtained calculated from relevant data within the source. No variables were missing or unavailable, as referenced in [14].

^p^Data were obtained calculated from relevant data within the source. No variables were missing or unavailable, as referenced in [15].

^q^Data were obtained calculated from relevant data within the source. Real data for one variable (Others) is missing or unavailable, as referenced in [16].

^r^Data were obtained calculated from relevant data within the source. Real data for one variable (Others) is missing or unavailable, as referenced in [17].

^s^Data were obtained calculated from relevant data within the source. No variables were missing or unavailable, as referenced in [18].

^t^Data were obtained directly from the source. Real data for two variables (Asian and Hispanic) are missing or unavailable, as referenced in [19].

^u^Data were obtained calculated from relevant data within the source. No variables were missing or unavailable, as referenced in [20].

**Table 4.**  Chi-square analysis of gender and race distribution differences between Qwen-7B generated clinical EHRs and real-world data across 20 diseases (1000 cases per disease, N=20,000)

| **Diseases** | ${df}_{race}$ | $P_{race}$ | ${\chi^{2}}_{race}$ | ${df}_{gender}$ | $P_{gender}$ | ${\chi^{2}}_{gender}$ |
| --- | --- | --- | --- | --- | --- | --- |
| Amyotrophic Lateral Sclerosis | 2^b^ | <.001 | 131.446 | 1 | <.001 | 172.427 |
| Bacterial Pneumonia | 3^c^ | <.001 | 205.064 | 1 | <.001 | 368.588 |
| Colon cancer | 3^d^ | <.001 | 240.382 | 1 | <.001 | 390.292 |
| COVID-19 | 4^e^ | <.001 | 234.539 | 1 | <.001 | 189.981 |
| Hepatitis B | 3^f^ | <.001 | 194.654 | 1 | <.001 | 455.737 |
| HIV | 4^g^ | <.001 | 357.392 | 1 | <.001 | 181.597 |
| Huntington Disease | 3^h^ | <.001 | 283.252 | 1 | <.001 | 348.986 |
| Hypertension | 3^i^ | <.001 | 331.626 | 1 | <.001 | 449.350 |
| Lupus | 3^j^ | <.001 | 191.281 | 1 | <.001 | 76.329 |
| Major Depressive Disorder | 3^k^ | <.001 | 168.897 | 1 | <.001 | 172.891 |
| Multiple Myeloma | 4^l^ | <.001 | 264.298 | 1 | <.001 | 453.114 |
| Multiple Sclerosis | 4^m^ | <.001 | 184.469 | 1 | <.001 | 131.636 |
| Preeclampsia | 3^n^ | <.001 | 281.625 | N/A^a^ | N/A^a^ | N/A^a^ |
| Prostate cancer | 4^o^ | <.001 | 84.030 | N/A^a^ | N/A^a^ | N/A^a^ |
| Rheumatoid Arthritis | 4^p^ | <.001 | 289.781 | 1 | <.001 | 216.107 |
| Scaecoidosis | 3^q^ | <.001 | 91.397 | 1 | <.001 | 23.510 |
| Syphilis | 3^r^ | <.001 | 307.585 | 1 | <.001 | 205.752 |
| Takotsubo cardiomyopathy | 4^s^ | <.001 | 230.645 | 1 | <.001 | 119.943 |
| Tricuspid Endocarditis | 2^t^ | <.001 | 83.407 | 1 | <.001 | 328.679 |
| Tuberculosis | 4^u^ | <.001 | 433.147 | 1 | <.001 | 128.675 |

^a^There are N/A values where we are unable to assess statistical significance when the conditions are present in only one gender.

^b^Data were obtained directly from the source. Real data for two variables(Asian and Hispanic) are missing or unavailable, as referenced in [1].

^c^Data were obtained directly from the source. Real data for one variables(Asian) is missing or unavailable, as referenced in [2].

^d^Data were obtained calculated from relevant data within the source. Real data for one variables (Others) is missing or unavailable, as referenced in [3].

^e^Data were obtained directly from the source. No variables were missing or unavailable, as referenced in [4].

^f^Data were obtained calculated from relevant data within the source. Real data for one variables (Others) is missing or unavailable, as referenced in [5].

^g^Data were obtained calculated from relevant data within the source. No variables were missing or unavailable, as referenced in [6].

^h^Data were obtained directly from the source. Real data for one variable (Others) is missing or unavailable, as referenced in [7].

^i^Data were obtained calculated from relevant data within the source. Real data for one variable (Others) is missing or unavailable, as referenced in [8].

^j^Data were obtained calculated from relevant data within the source. Real data for one variables (Others) is missing or unavailable, as referenced in [9].

^k^Data were obtained directly from the source. Real data for one variable(Others) is missing or unavailable, as referenced in [10].

^l^Data were obtained calculated from relevant data within the source. No variables were missing or unavailable, as referenced in [11].

^m^Data were obtained calculated from relevant data within the source. No variables were missing or unavailable, as referenced in [12].

^n^Data were obtained calculated from relevant data within the source. Real data for one variable (Others) is missing or unavailable, as referenced in [13].

^o^Data were obtained calculated from relevant data within the source. No variables were missing or unavailable, as referenced in [14].

^p^Data were obtained calculated from relevant data within the source. No variables were missing or unavailable, as referenced in [15].

^q^Data were obtained calculated from relevant data within the source. Real data for one variable (Others) is missing or unavailable, as referenced in [16].

^r^Data were obtained calculated from relevant data within the source. Real data for one variable (Others) is missing or unavailable, as referenced in [17].

^s^Data were obtained calculated from relevant data within the source. No variables were missing or unavailable, as referenced in [18].

^t^Data were obtained directly from the source. Real data for two variables (Asian and Hispanic) are missing or unavailable, as referenced in [19].

^u^Data were obtained calculated from relevant data within the source. No variables were missing or unavailable, as referenced in [20].

**Table 5.** Chi-square analysis of gender and race distribution differences between Qwen-14B generated clinical EHRs and real-world data across 20 diseases (1000 cases per disease, N=20,000)

| **Diseases** | ${df}_{race}$ | $P_{race}$ | ${\chi^{2}}_{race}$ | ${df}_{gender}$ | $P_{gender}$ | ${\chi^{2}}_{gender}$ |
| --- | --- | --- | --- | --- | --- | --- |
| Amyotrophic Lateral Sclerosis | 2^b^ | <.001 | 236.764 | 1 | <.001 | 577.974 |
| Bacterial Pneumonia | 3^c^ | <.001 | 148.890 | 1 | <.001 | 597.137 |
| Colon cancer | 3^d^ | <.001 | 281.615 | 1 | <.001 | 530.988 |
| COVID-19 | 4^e^ | <.001 | 391.818 | 1 | <.001 | 453.258 |
| Hepatitis B | 3^f^ | <.001 | 787.202 | 1 | <.001 | 485.871 |
| HIV | 4^g^ | <.001 | 587.436 | 1 | <.001 | 198.774 |
| Huntington Disease | 3^h^ | <.001 | 261.817 | 1 | <.001 | 138.390 |
| Hypertension | 3^i^ | <.001 | 501.640 | 1 | <.001 | 558.824 |
| Lupus | 3^j^ | <.001 | 72.455 | 1 | <.001 | 94.037 |
| Major Depressive Disorder | 3^k^ | <.001 | 222.126 | 1 | <.001 | 420.224 |
| Multiple Myeloma | 4^l^ | <.001 | 294.127 | 1 | <.001 | 562.567 |
| Multiple Sclerosis | 4^m^ | <.001 | 176.305 | 1 | <.001 | 239.453 |
| Preeclampsia | 3^n^ | <.001 | 340.011 | N/A^a^ | N/A^a^ | N/A^a^ |
| Prostate cancer | 4^o^ | <.001 | 100.015 | N/A^a^ | N/A^a^ | N/A^a^ |
| Rheumatoid Arthritis | 4^p^ | <.001 | 304.240 | 1 | <.001 | 246.222 |
| Scaecoidosis | 3^q^ | <.001 | 795.439 | 1 | <.001 | 7.7628 |
| Syphilis | 3^r^ | <.001 | 276.836 | 1 | <.001 | 219.714 |
| Takotsubo cardiomyopathy | 4^s^ | <.001 | 306.753 | 1 | <.001 | 119.286 |
| Tricuspid Endocarditis | 2^t^ | <.001 | 227.932 | 1 | <.001 | 366.961 |
| Tuberculosis | 4^u^ | <.001 | 582.259 | 1 | <.001 | 436.566 |

^a^There are N/A values where we are unable to assess statistical significance when the conditions are present in only one gender.

^b^Data were obtained directly from the source. Real data for two variables(Asian and Hispanic) are missing or unavailable, as referenced in [1].

^c^Data were obtained directly from the source. Real data for one variables(Asian) is missing or unavailable, as referenced in [2].

^d^Data were obtained calculated from relevant data within the source. Real data for one variables (Others) is missing or unavailable, as referenced in [3].

^e^Data were obtained directly from the source. No variables were missing or unavailable, as referenced in [4].

^f^Data were obtained calculated from relevant data within the source. Real data for one variables (Others) is missing or unavailable, as referenced in [5].

^g^Data were obtained calculated from relevant data within the source. No variables were missing or unavailable, as referenced in [6].

^h^Data were obtained directly from the source. Real data for one variable (Others) is missing or unavailable, as referenced in [7].

^i^Data were obtained calculated from relevant data within the source. Real data for one variable (Others) is missing or unavailable, as referenced in [8].

^j^Data were obtained calculated from relevant data within the source. Real data for one variables (Others) is missing or unavailable, as referenced in [9].

^k^Data were obtained directly from the source. Real data for one variable(Others) is missing or unavailable, as referenced in [10].

^l^Data were obtained calculated from relevant data within the source. No variables were missing or unavailable, as referenced in [11].

^m^Data were obtained calculated from relevant data within the source. No variables were missing or unavailable, as referenced in [12].

^n^Data were obtained calculated from relevant data within the source. Real data for one variable (Others) is missing or unavailable, as referenced in [13].

^o^Data were obtained calculated from relevant data within the source. No variables were missing or unavailable, as referenced in [14].

^p^Data were obtained calculated from relevant data within the source. No variables were missing or unavailable, as referenced in [15].

^q^Data were obtained calculated from relevant data within the source. Real data for one variable (Others) is missing or unavailable, as referenced in [16].

^r^Data were obtained calculated from relevant data within the source. Real data for one variable (Others) is missing or unavailable, as referenced in [17].

^s^Data were obtained calculated from relevant data within the source. No variables were missing or unavailable, as referenced in [18].

^t^Data were obtained directly from the source. Real data for two variables (Asian and Hispanic) are missing or unavailable, as referenced in [19].

^u^Data were obtained calculated from relevant data within the source. No variables were missing or unavailable, as referenced in [20].

**Table 6.** Chi-square analysis of gender and race distribution differences between Yi-6B generated clinical EHRs and real-world data across 20 diseases (1000 cases per disease, N=20,000)

| **Diseases** | ${df}_{race}$ | $P_{race}$ | ${\chi^{2}}_{race}$ | ${df}_{gender}$ | $P_{gender}$ | ${\chi^{2}}_{gender}$ |
| --- | --- | --- | --- | --- | --- | --- |
| Amyotrophic Lateral Sclerosis | 2^b^ | <.001 | 77.386 | 1 | <.001 | 279.100 |
| Bacterial Pneumonia | 3^c^ | <.001 | 304.916 | 1 | <.001 | 248.079 |
| Colon cancer | 3^d^ | <.001 | 302.049 | 1 | <.001 | 296.366 |
| COVID-19 | 4^e^ | <.001 | 480.604 | 1 | <.001 | 85.401 |
| Hepatitis B | 3^f^ | <.001 | 355.138 | 1 | <.001 | 219.524 |
| HIV | 4^g^ | <.001 | 602.475 | 1 | <.001 | 77.029 |
| Huntington Disease | 3^h^ | <.001 | 242.969 | 1 | <.001 | 168.860 |
| Hypertension | 3^i^ | <.001 | 368.186 | 1 | <.001 | 360.275 |
| Lupus | 3^j^ | <.001 | 386.654 | 1 | <.001 | 12.839 |
| Major Depressive Disorder | 3^k^ | <.001 | 273.493 | 1 | .4 | 0.703 |
| Multiple Myeloma | 4^l^ | <.001 | 528.872 | 1 | <.001 | 255.051 |
| Multiple Sclerosis | 4^m^ | <.001 | 422.455 | 1 | <.001 | 11.598 |
| Preeclampsia | 3^n^ | <.001 | 150.442 | N/A^a^ | N/A^a^ | N/A^a^ |
| Prostate cancer | 4^o^ | <.001 | 358.545 | N/A^a^ | N/A^a^ | N/A^a^ |
| Rheumatoid Arthritis | 4^p^ | <.001 | 473.266 | 1 | .7 | 0.111 |
| Scaecoidosis | 3^q^ | <.001 | 146.728 | 1 | $.3$ | 1.106 |
| Syphilis | 3^r^ | <.001 | 501.369 | 1 | <.001 | 80.935 |
| Takotsubo cardiomyopathy | 4^s^ | <.001 | 498.747 | 1 | $.5$ | 0.497 |
| Tricuspid Endocarditis | 2^t^ | <.001 | 324.381 | 1 | <.001 | 55.922 |
| Tuberculosis | 4^u^ | <.001 | 958.124 | 1 | <.001 | 87.432 |

^a^There are N/A values where we are unable to assess statistical significance when the conditions are present in only one gender.

^b^Data were obtained directly from the source. Real data for two variables(Asian and Hispanic) are missing or unavailable, as referenced in [1].

^c^Data were obtained directly from the source. Real data for one variables(Asian) is missing or unavailable, as referenced in [2].

^d^Data were obtained calculated from relevant data within the source. Real data for one variables (Others) is missing or unavailable, as referenced in [3].

^e^Data were obtained directly from the source. No variables were missing or unavailable, as referenced in [4].

^f^Data were obtained calculated from relevant data within the source. Real data for one variables (Others) is missing or unavailable, as referenced in [5].

^g^Data were obtained calculated from relevant data within the source. No variables were missing or unavailable, as referenced in [6].

^h^Data were obtained directly from the source. Real data for one variable (Others) is missing or unavailable, as referenced in [7].

^i^Data were obtained calculated from relevant data within the source. Real data for one variable (Others) is missing or unavailable, as referenced in [8].

^j^Data were obtained calculated from relevant data within the source. Real data for one variables (Others) is missing or unavailable, as referenced in [9].

^k^Data were obtained directly from the source. Real data for one variable(Others) is missing or unavailable, as referenced in [10].

^l^Data were obtained calculated from relevant data within the source. No variables were missing or unavailable, as referenced in [11].

^m^Data were obtained calculated from relevant data within the source. No variables were missing or unavailable, as referenced in [12].

^n^Data were obtained calculated from relevant data within the source. Real data for one variable (Others) is missing or unavailable, as referenced in [13].

^o^Data were obtained calculated from relevant data within the source. No variables were missing or unavailable, as referenced in [14].

^p^Data were obtained calculated from relevant data within the source. No variables were missing or unavailable, as referenced in [15].

^q^Data were obtained calculated from relevant data within the source. Real data for one variable (Others) is missing or unavailable, as referenced in [16].

^r^Data were obtained calculated from relevant data within the source. Real data for one variable (Others) is missing or unavailable, as referenced in [17].

^s^Data were obtained calculated from relevant data within the source. No variables were missing or unavailable, as referenced in [18].

^t^Data were obtained directly from the source. Real data for two variables (Asian and Hispanic) are missing or unavailable, as referenced in [19].

^u^Data were obtained calculated from relevant data within the source. No variables were missing or unavailable, as referenced in [20].

**Table 7.** Chi-square analysis of gender and race distribution differences between Yi-34B generated clinical EHRs and real-world data across 20 diseases (1000 cases per disease, N=20,000)

| **Diseases** | ${df}_{race}$ | $P_{race}$ | ${\chi^{2}}_{race}$ | ${df}_{gender}$ | $P_{gender}$ | ${\chi^{2}}_{gender}$ |
| --- | --- | --- | --- | --- | --- | --- |
| Amyotrophic Lateral Sclerosis | 2^b^ | <.001 | 206.432 | 1 | <.001 | 525.982 |
| Bacterial Pneumonia | 3^c^ | <.001 | 95.614 | 1 | <.001 | 429.002 |
| Colon cancer | 3^d^ | <.001 | 238.402 | 1 | <.001 | 446.441 |
| COVID-19 | 4^e^ | <.001 | 232.263 | 1 | <.001 | 230.314 |
| Hepatitis B | 3^f^ | <.001 | 352.038 | 1 | <.001 | 304.898 |
| HIV | 4^g^ | <.001 | 696.780 | 1 | <.001 | 103.241 |
| Huntington Disease | 3^h^ | <.001 | 326.643 | 1 | <.001 | 190.158 |
| Hypertension | 3^i^ | <.001 | 885.430 | 1 | <.001 | 459.481 |
| Lupus | 3^j^ | <.001 | 192.494 | 1 | <.001 | 110.092 |
| Major Depressive Disorder | 3^k^ | <.001 | 256.655 | 1 | <.001 | 61.254 |
| Multiple Myeloma | 4^l^ | <.001 | 237.505 | 1 | <.001 | 378.608 |
| Multiple Sclerosis | 4^m^ | <.001 | 274.402 | 1 | .2 | 1.67173 |
| Preeclampsia | 3^n^ | <.001 | 603.603 | N/A^a^ | N/A^a^ | N/A^a^ |
| Prostate cancer | 4^o^ | <.001 | 140.016 | N/A^a^ | N/A^a^ | N/A^a^ |
| Rheumatoid Arthritis | 4^p^ | <.001 | 241.851 | 1 | <.001 | 221.446 |
| Scaecoidosis | 3^q^ | <.001 | 673.339 | 1 | <.001 | 426.132 |
| Syphilis | 3^r^ | <.001 | 426.897 | 1 | <.001 | 175.519 |
| Takotsubo cardiomyopathy | 4^s^ | <.001 | 145.145 | 1 | <.001 | 134.050 |
| Tricuspid Endocarditis | 2^t^ | <.001 | 49.138 | 1 | <.001 | 412.600 |
| Tuberculosis | 4^u^ | <.001 | 767.084 | 1 | <.001 | 216.460 |

^a^There are N/A values where we are unable to assess statistical significance when the conditions are present in only one gender.

^b^Data were obtained directly from the source. Real data for two variables(Asian and Hispanic) are missing or unavailable, as referenced in [1].

^c^Data were obtained directly from the source. Real data for one variables(Asian) is missing or unavailable, as referenced in [2].

^d^Data were obtained calculated from relevant data within the source. Real data for one variables (Others) is missing or unavailable, as referenced in [3].

^e^Data were obtained directly from the source. No variables were missing or unavailable, as referenced in [4].

^f^Data were obtained calculated from relevant data within the source. Real data for one variables (Others) is missing or unavailable, as referenced in [5].

^g^Data were obtained calculated from relevant data within the source. No variables were missing or unavailable, as referenced in [6].

^h^Data were obtained directly from the source. Real data for one variable (Others) is missing or unavailable, as referenced in [7].

^i^Data were obtained calculated from relevant data within the source. Real data for one variable (Others) is missing or unavailable, as referenced in [8].

^j^Data were obtained calculated from relevant data within the source. Real data for one variables (Others) is missing or unavailable, as referenced in [9].

^k^Data were obtained directly from the source. Real data for one variable(Others) is missing or unavailable, as referenced in [10].

^l^Data were obtained calculated from relevant data within the source. No variables were missing or unavailable, as referenced in [11].

^m^Data were obtained calculated from relevant data within the source. No variables were missing or unavailable, as referenced in [12].

^n^Data were obtained calculated from relevant data within the source. Real data for one variable (Others) is missing or unavailable, as referenced in [13].

^o^Data were obtained calculated from relevant data within the source. No variables were missing or unavailable, as referenced in [14].

^p^Data were obtained calculated from relevant data within the source. No variables were missing or unavailable, as referenced in [15].

^q^Data were obtained calculated from relevant data within the source. Real data for one variable (Others) is missing or unavailable, as referenced in [16].

^r^Data were obtained calculated from relevant data within the source. Real data for one variable (Others) is missing or unavailable, as referenced in [17].

^s^Data were obtained calculated from relevant data within the source. No variables were missing or unavailable, as referenced in [18].

^t^Data were obtained directly from the source. Real data for two variables (Asian and Hispanic) are missing or unavailable, as referenced in [19].

^u^Data were obtained calculated from relevant data within the source. No variables were missing or unavailable, as referenced in [20].

References

1. Mehta P, Raymond J, Zhang Y, Punjani R, Han M, Larson T, Muravov O, Lyles RH, Horton DK. Prevalence of amyotrophic lateral sclerosis in the United States, 2018. Amyotrophic Lateral Sclerosis and Frontotemporal Degeneration. 2023;24(7-8):702–708. doi:10.1080/21678421.2023.2245858.
2. Burton DC, Flannery B, Bennett NM, Farley MM, Gershman K, Harrison LH, Lynfield R, Petit S, Reingold AL, Schaffner W, Thomas A, Plikaytis BD, Rose CE, Whitney CG, Schuchat A. Socioeconomic and racial/ethnic disparities in the incidence of bacteremic pneumonia among US adults. Am J Public Health. 2009;100(10):1904–1911. doi:10.2105/AJPH.2009.181313.
3. Siegel RL, Wagle NS, Cercek A, Smith RA, Jemal A. Colorectal cancer statistics, 2023. CA Cancer J Clin. 2023;73(3):233–254. doi:10.3322/caac.21772.
4. Centers for Disease Control and Prevention. CDC COVID Data Tracker. 2024. URL:<https://covid.cdc.gov/covid-data-tracker> Accessed April 30, 2024.
5. Kruszon-Moran D, Paulose-Ram R, Martin CB, Barker LK, McQuillan G. Prevalence and Trends in Hepatitis B Virus Infection in the United States, 2015-2018. NCHS Data Brief. 2020 Mar;(361):1-8. PMID: 32487291.
6. CDC. HIV in the United States by Race/Ethnicity: HIV Diagnoses. 2024. URL:<https://www.cdc.gov/hiv/group/racialethnic/other-races/diagnoses.html> Accessed April 29, 2024.
7. Bruzelius E, Scarpa J, Zhao Y, Basu S, Faghmous JH, Baum A. Huntington’s disease in the United States: Variation by demographic and socioeconomic factors. Mov Disord. 2024;34(6):858–865. doi:10.1002/mds.27653. Accessed March 25, 2024.
8. Whelton PK, Carey RM, Aronow WS, Casey DE, Collins KJ, Dennison Himmelfarb C, DePalma SM, Gidding S, Jamerson KA, Jones DW, MacLaughlin EJ, Muntner P, Ovbiagele B, Smith SC, Spencer CC, Stafford RS, Taler SJ, Thomas RJ, Williams KA, Williamson JD, Wright JT. 2017 ACC/AHA/AAPA/ABC/ACPM/AGS/APhA/ASH/ASPC/NMA/PCNA guideline for the prevention, detection, evaluation, and management of high blood pressure in adults: Executive summary: A report of the American College of Cardiology/American Heart Association Task Force on Clinical Practice Guidelines. Hypertension. 2018;138(17):426–483. doi:10.1161/CIR.0000000000000597.
9. Izmirly PM, Ferucci ED, Somers EC, Wang L, Lim SS, Drenkard C, Dall’Era M, McCune WJ, Gordon C, Helmick C, Parton H. Incidence rates of systemic lupus erythematosus in the USA: Estimates from a meta-analysis of the Centers for Disease Control and Prevention National Lupus Registries. Lupus Science & Medicine. 2024;8(1):e000614. doi:10.1136/lupus-2021-000614. Accessed April 29, 2024.
10. National Institute of Mental Health (NIMH). Major Depression. URL:<https://www.nimh.nih.gov/health/statistics/major-depression.> Accessed April 30, 2024.
11. Centers for Disease Control and Prevention. United States Cancer Statistics: Data Visualizations. 2024. URL:<https://gis.cdc.gov/grasp/USCS/DataViz.html> Accessed April 30, 2024.
12. Hittle M, Culpepper WJ, Langer-Gould A, Marrie RA, Cutter GR, Kaye WE, Wagner L, Topol B, LaRocca NG, Nelson LM, Wallin MT. Population-based estimates for the prevalence of multiple sclerosis in the United States by race, ethnicity, age, sex, and geographic region. JAMA Neurol. 2023;80(7):693–701. doi:10.1001/jamaneurol.2023.1135.
13. Fingar KR, Mabry-Hernandez I, Ngo-Metzger Q, Wolff T, Steiner CA, Elixhauser A. Delivery hospitalizations involving preeclampsia and eclampsia, 2005–2014. In: Healthcare Cost and Utilization Project (HCUP) Statistical Briefs. Agency for Healthcare Research and Quality (US). 2024. URL:<http://www.ncbi.nlm.nih.gov/books/NBK442039/> Accessed April 29, 2024.
14. Siegel DA. Prostate cancer incidence and survival, by stage and race/ethnicity — United States, 2001–2017. MMWR Morb Mortal Wkly Rep. 2024;69:1473–1480. doi:10.15585/mmwr.mm6941a1. Accessed April 29, 2024.
15. Kawatkar AA, Gabriel SE, Jacobsen SJ. Secular trends in the incidence and prevalence of rheumatoid arthritis within members of an integrated health care delivery system. Arthritis Rheum. 2018;39(3):541–549. doi:10.1007/s00296-018-04235-y.
16. Baughman RP, Field S, Costabel U, Crystal RG, Culver DA, Drent M, Judson MA, Wolff G. Sarcoidosis in America: Analysis Based on Health Care Use. Annals of the American Thoracic Society. 2024;13(8):1244–1252. doi:10.1513/AnnalsATS.201511-760OC.
17. CDC. Cases of STDs Reported by Disease and State, 2021. 2024. URL:<https://www.cdc.gov/std/statistics/2021/tables/15.htm> Accessed January 28, 2024.
18. Zaghlol R, Dey AK, Desale S, Barac A. Racial differences in takotsubo-cardiomyopathy outcomes in a large nationwide sample. Eur Heart J Qual Care Clin Outcomes. 2024;7(3):1056–1063. doi:10.1002/ehf2.12664.
19. Khan MZ. Racial and gender trends in infective endocarditis related deaths in the United States (2004-2017). Am J Cardiol. 2020;129:125–126. doi:10.1016/j.amjcard.2020.05.037.
20. CDC. Reported TB in the US 2020. 2024. URL:<https://www.cdc.gov/tb/statistics/reports/2020/table20.htm> Accessed April 29, 2024.
