# Supplementary material for: Evaluation and Bias Analysis of Large Language Models in Generating Synthetic Electronic Health Records: Comparative Study
Source: J Med Internet Res. 2025 May 12;27:e65317. doi: 10.2196/65317 (PMC12107208; doi:10.2196/65317)
Supplement: Multimedia Appendix 3 [file jmir_v27i1e65317_app3.docx]

## Multimedia Appendix 3: Racial Bias Distribution

**Table 1.** Racial Bias Distribution in the Qwen Model for Parameter Sizes of 1.8B, 7B, and 14B.

| **Diseases** | **Race** | **Qwen-1.8B Ratio and SPD**  **(n=1000,%)** | **Qwen-7B Ratio and SPD**  **(n=1000,%)** | **Qwen-14B Ratio and SPD**  **(n=1000,%)** | **Actual Ratio**  **(%)** |
| --- | --- | --- | --- | --- | --- |
| Amyotrophic Lateral Sclerosis | Not Available | 37.8 | 9.2 | 1.8 | 26.8 |
|  | Black | 9.2  (+2.7) | 2.5  (-4.0) | 6.5  (0.0) | 6.5 |
|  | White | 46.8  (**-19.9**) | 87.5  (**+20.8**) | 87.4  (**+20.7**) | 66.7 |
|  | Hispanic | 4.3  (+4.3) | 0.6  (+0.6) | 0.2  (+0.2) | 0.0^a^ |
|  | Asian | 1.9  (+1.9) | 0.2  (+0.2) | 4.1  (+4.1) | 0.0^a^ |
| Bacterial Pneumonia | Not Available | 34.2 | 9.4 | 20.4 | 11.0 |
|  | Black | 15.3  (-9.7) | 4.4  (**-20.6**) | 8.3  (**-16.7**) | 25.0 |
|  | White | 42.5  (**-18.5**) | 84.4  (**+23.4**) | 70.9  (+9.9) | 61.0 |
|  | Hispanic | 7.0  (+4.0) | 0.2  (-2.8) | 0.0  (-3.0) | 3.0 |
|  | Asian | 1.0  (+1.0) | 1.6  (+1.6) | 0.4  (+0.4) | 0.0^b^ |
| Colon cancer | Not Available | 29.0 | 7.0 | 10.7 | 0.0^c^ |
|  | Black | 25.6  (**+10.0**) | 6.7  (-8.9) | 5.5  (**-10.1**) | 15.6 |
|  | White | 39.5  (**-23.1**) | 83.9  (**+21.3**) | 83.8  (**+21.2**) | 62.6 |
|  | Hispanic | 3.9  (**-14.7**) | 0.2  (**-18.4**) | 0.0  (**-18.6**) | 18.6 |
|  | Asian | 2  (-3.2) | 2.2  (-3.0) | 0.0  (-5.2) | 5.2 |
| COVID-19 | Not Available | 37.0 | 12.9 | 28.4 | 1.3 |
|  | Black | 14.8  (+1.1) | 7.0  (-6.7) | 4.2  (-9.5) | 13.7 |
|  | White | 40.3  (**-26.7**) | 74.4  (+7.4) | 61.3  (-5.7) | 67.0 |
|  | Hispanic | 4.5  (**-10.4**) | 1.4  (**-13.2**) | 2.4  (**-12.5**) | 14.9 |
|  | Asian | 3.4  (+0.3) | 4.3  (+1.2) | 3.7  (+0.6) | 3.1 |
| Hepatitis B | Not Available | 36.9 | 14.4 | 4.4 | 0.0^d^ |
|  | Black | 16.9  (**-13.5**) | 13.7  (**-16.7**) | 0.0  (**-30.4**) | 30.4 |
|  | White | 30.8  (+2.6) | 38.9  (**+10.7**) | 11.2  (**-17.0**) | 28.2 |
|  | Hispanic | 4.4  (**-12.1**) | 0.4  (**-16.1**) | 0.0  (**-16.5**) | 16.5 |
|  | Asian | 11.0  (**-18.0**) | 32.6  (+3.6) | 84.4  (**+55.4**) | 29.0 |
| HIV | Not Available | 25.3 | 6.6 | 4.8 | 3.2 |
|  | Black | 51.8  (**+10.1**) | 59.8  (**+18.1**) | 88.9  (**+47.2**) | 41.7 |
|  | White | 20.8  (-3.7) | 33.4  (+8.9) | 6.3  (**-18.2**) | 24.5 |
|  | Hispanic | 1.7  (**-26.9**) | 0.1  (**-28.5**) | 0.0  (**-28.6**) | 28.6 |
|  | Asian | 0.4  (-1.6) | 0.1  (-1.9) | 0.0  (-2.0) | 2.0 |
| Huntington Disease | Not Available | 44.9 | 4.7 | 1.8 | 0.0^e^ |
|  | Black | 16.8  (+2.8) | 1.2  (**-12.8**) | 1.2  (**-12.8**) | 14.0 |
|  | White | 31.8  (**-38.3**) | 93.9  (**+23.8**) | 95.1  (**+25.0**) | 70.1 |
|  | Hispanic | 4.5  (-8.2) | 0.2  (**-12.5**) | 0.5  (**-12.2**) | 12.7 |
|  | Asian | 2.0  (-1.3) | 0.0  (-3.3) | 1.4  (-1.9) | 3.3 |
| Hypertension | Not Available | 28.8 | 4.5 | 7.8 | 0.0^f^ |
|  | Black | 27.8  (**+13.1**) | 24.4  (+9.7) | 49.7  (**+35.0**) | 14.7 |
|  | White | 35.1  (**-18.5**) | 70.1  (**+16.5**) | 42.5  (**-11.1**) | 53.6 |
|  | Hispanic | 6.7  (**-10.3**) | 0.5  (**-16.5**) | 0.0  (**-17.0**) | 17.0 |
|  | Asian | 1.6  (**-14.4**) | 0.5  (**-15.5**) | 0.0  (**-16.0**) | 16.0 |
| Lupus | Not Available | 30.0 | 7.8 | 6.3 | 0.0^g^ |
|  | Black | 30.6  (+2.2) | 17.0  (**-11.4**) | 28.8  (+0.4) | 28.4 |
|  | White | 32.5  (**-15.6**) | 70.5  (+22.4) | 53.4  (+5.3) | 48.1 |
|  | Hispanic | 3.3  (**-15.2**) | 3.1  (**-15.4**) | 11.5  (-7.0) | 18.5 |
|  | Asian | 3.6  (-2.4) | 1.6  (-4.4) | 0.0  (-6.0) | 6.0 |
| Major Depressive Disorder | Not Available | 44.6 | 7.5 | 2.4 | 0.0^h^ |
|  | Black | 6.8  (-3.7) | 10.6  (+0.1) | 6.5  (-4.0) | 10.5 |
|  | White | 37.9  (**-28.8**) | 78.8  (**+12.1**) | 88.7  (**+22.0**) | 66.7 |
|  | Hispanic | 7.1  (**-12.0**) | 0.7  (**-18.4**) | 0.3  (**-18.8**) | 19.1 |
|  | Asian | 3.6  (-0.1) | 2.4  (-1.3) | 2.1  (-1.6) | 3.7 |
| Multiple Myeloma | Not Available | 29.2 | 6.8 | 16.7 | 0.6 |
|  | Black | 17.8  (-3.4) | 6.5  (**-14.7**) | 13.8  (-7.4) | 21.2 |
|  | White | 44.3  (**-21.2**) | 85.9  (**+20.4**) | 69.5  (+4.0) | 65.5 |
|  | Hispanic | 4.9  (-4.8) | 0.3  (-9.4) | 0  (-9.7) | 9.7 |
|  | Asian | 3.8  (+0.8) | 0.5  (-2.5) | 0  (-3.0) | 3.0 |
| Multiple Sclerosis | Not Available | 24.6 | 8.8 | 5.7 | 0.0^i^ |
|  | Black | 19.3  (+8.5) | 6.6  (-4.2) | 10.2  (-0.6) | 10.8 |
|  | White | 49.9  (**-27.7**) | 83.3  (+5.7) | 84.1  (+6.5) | 77.6 |
|  | Hispanic | 5.0  (-2.2) | 0.4  (-6.8) | 0.0  (-7.2) | 7.2 |
|  | Asian | 1.2  (-3.3) | 0.9  (-3.6) | 0.0  (-4.5) | 4.5 |
| Preeclampsia | Not Available | 33.3 | 4.8 | 9.1 | 0.0^j^ |
|  | Black | 26.7  (+8.7) | 43.2  (**+25.2**) | 53.3  (**+35.3**) | 18.0 |
|  | White | 30.8  (**-22.4**) | 49.8  (-3.4) | 31.7  (**-21.5**) | 53.2 |
|  | Hispanic | 6.0  (**-12.6**) | 1.5  (**-17.1**) | 2.6  (**-16.0**) | 18.6 |
|  | Asian | 3.2  (-2.6) | 0.7  (-5.1) | 3.3  (-2.5) | 5.8 |
| Prostate cancer | Not Available | 34.4 | 5.1 | 0.8 | 6.0 |
|  | Black | 20.4  (+5.8) | 18.4  (+3.8) | 17.5  (+2.9) | 14.6 |
|  | White | 42.4  (**-32.0**) | 76.0  (+1.6) | 76.5  (+2.1) | 74.4 |
|  | Hispanic | 1.8  (-4.6) | 0.1  (-6.3) | 0.0  (-6.4) | 6.4 |
|  | Asian | 1.0  (-1.0) | 0.4  (-1.6) | 0.0  (-2.0) | 2.0 |
| Rheumatoid Arthritis | Not Available | 34.4 | 10.4 | 10.6 | 8.6 |
|  | Black | 12.1  (+0.8) | 5.1  (-6.2) | 6.7  (-4.6) | 11.3 |
|  | White | 43.8  (-9.9) | 81.4  (**+27.7**) | 81.6  (**+27.9**) | 53.7 |
|  | Hispanic | 5.9  (**-15.8**) | 0.5  (**-21.2**) | 1.1  (**-20.6**) | 21.7 |
|  | Asian | 3.8  (-1.0) | 2.6  (-2.2) | 0.0  (-4.8) | 4.8 |
| Scaecoidosis | Not Available | 29.9 | 3.2 | 1.3 | 0.0^k^ |
|  | Black | 13.0  (**-20.2**) | 34.7  (+1.5) | 92.6  (**+59.4**) | 33.2 |
|  | White | 50.5  (-7.4) | 61.7  (+3.8) | 6.1  (**-51.8**) | 57.9 |
|  | Hispanic | 3.8  (-4.3) | 0.0  (-8.1) | 0.0  (-8.1) | 8.1 |
|  | Asian | 2.8  (+0.6) | 0.4  (-1.8) | 0.0  (-2.2) | 2.2 |
| Syphilis | Not Available | 28.6 | 8.9 | 36.9 | 0.0^l^ |
|  | Black | 35.1  (-2.9) | 20.8  (**-17.2**) | 6.1  (**-31.9**) | 38.0 |
|  | White | 34.3  (-4.4) | 68.3  (**+29.6**) | 54.8  (**+16.1**) | 38.7 |
|  | Hispanic | 1.6  (**-21.7**) | 1.6  (**-21.7**) | 2.2  (**-21.1**) | 23.3 |
|  | Asian | 0.4  (-1.7) | 0.4  (-1.7) | 0.0  (-2.1) | 2.1 |
| Takotsubo cardiomyopathySyphilis | Not Available | 64.2 | 14.3 | 6.5 | 0.7 |
|  | Black | 2.3  (-5.0) | 1.3  (-6.0) | 0.0  (-7.3) | 7.3 |
|  | White | 10.3  (**-71.2**) | 75.4  (-6.1) | 70.1  (**-11.4**) | 81.5 |
|  | Hispanic | 1.0  (-4.7) | 0.2  (-5.5) | 0.0  (-5.7) | 5.7 |
|  | Asian | 22.2  (**+17.3**) | 8.8  (+3.9) | 23.4  (**+18.5**) | 4.9 |
| Tricuspid Endocarditis | Not Available | 32.8 | 13.2 | 26.4 | 3.0 |
|  | Black | 14.1  (-1.6) | 9.1  (-6.6) | 10.0  (-5.7) | 15.7 |
|  | White | 41.1  (**-40.2**) | 74.8  (-6.5) | 60.4  (**-20.9**) | 81.3 |
|  | Hispanic | 9.1  (+9.1) | 0.3  (+0.3) | 2.7  (+2.7) | 0.0^m^ |
|  | Asian | 2.9  (+2.9) | 2.6  (+2.6) | 0.5  (+0.5) | 0.0^m^ |
| Tuberculosis | Not Available | 39.2 | 18.6 | 15.7 | 3.0 |
|  | Black | 25.1  (+5.7) | 23.2  (+3.8) | 35.0  (**+15.6**) | 19.4 |
|  | White | 30.8  (**+19.5**) | 22.1  (**+10.8**) | 33.4  (**+22.1**) | 11.3 |
|  | Hispanic | 3.0  (**-26.2**) | 0.3  (**-28.9**) | 12.2  (**-17.0**) | 29.2 |
|  | Asian | 1.9  (**-35.2**) | 35.8  (-1.3) | 3.7  (**-33.4**) | 37.1 |

^a^Data were obtained directly from the source. Due to the absence of the two variables (Asian and Hispanic) , the default value is set to 0.0% , as referenced in [1].

^b^Data were obtained directly from the source. Due to the absence of the one variable (Asian), the default value is set to 0.0% , as referenced in [2].

^c^Data were obtained calculated from relevant data within the source. Due to the absence of one variable (Not Available), the default value is set to 0.0% , as referenced in [3].

^d^Data were obtained calculated from relevant data within the source. Due to the absence of one variable (Not Available), the default value is set to 0.0% , as referenced in [5].

^e^Data were obtained directly from the source. Due to the absence of one variable (Not Available), the default value is set to 0.0% , as referenced in [7].

^f^Data were obtained calculated from relevant data within the source. Due to the absence of one variable (Not Available), the default value is set to 0.0% , as referenced in [8].

^g^Data were obtained calculated from relevant data within the source. Due to the absence of one variable (Not Available), the default value is set to 0.0% , as referenced in [9].

^h^Data were obtained directly from the source. Due to the absence of one variable (Not Available), the default value is set to 0.0% , as referenced in [10].

^i^Data were obtained calculated from relevant data within the source.Due to the variable (Not Available) is 0.000134259. For clarity and ease of presentation, this value is rounded and also set as the default to 0.0% , as referenced in [11].

^j^Data were obtained calculated from relevant data within the source. Due to the absence of one variable (Not Available), the default value is set to 0.0% , as referenced in [13].

^k^Data were obtained calculated from relevant data within the source. Due to the absence of one variable (Not Available), the default value is set to 0.0% , as referenced in [16].

^l^Data were obtained calculated from relevant data within the source. Due to the absence of one variable (Not Available), the default value is set to 0.0% , as referenced in [17].

^m^Data were obtained directly from the source. Due to the absence of two variables (Asian and Hispanic), the default value is set to 0.0% , as referenced in [19].

**Table 2.** Racial Bias Distribution in the Llama2 Model for Parameter Sizes of 7B and 13B.

| **Diseases** | **Race** | **Llama2-7B**  **Ratio and SPD**  **(n=1000,%)** | **Llama2-13B Ratio and SPD**  **(n=1000,%)** | **Actual Ratio**  **(%)** |
| --- | --- | --- | --- | --- |
| Amyotrophic Lateral Sclerosis | Not Available | 0.5 | 9.5 | 26.8 |
|  | Black | 1.2  (-5.3) | 0.1  (-6.4) | 6.5 |
|  | White | 95.0  (**+28.3**) | 89.4  (**+22.7**) | 66.7 |
|  | Hispanic | 0.3  (+0.3) | 0.0  (0.0) | 0.0^a^ |
|  | Asian | 3.0  (+3.0) | 1.0  (+1.0) | 0.0^a^ |
| Bacterial Pneumonia | Not Available | 0.1 | 0.2 | 11.0 |
|  | Black | 6.2  (**-18.8**) | 2.0  (**-23.0**) | 25.0 |
|  | White | 87.7  (**+26.7**) | 97.4  (**+36.4**) | 61.0 |
|  | Hispanic | 3.0  (0.0) | 0.4  (-2.6) | 3.0 |
|  | Asian | 3.0  (+3.0) | 0.0  (0.0) | 0.0^b^ |
| Colon cancer | Not Available | 6.8 | 7.4 | 0.0^c^ |
|  | Black | 2.6  (**-13.0**) | 1.5  (**-14.1**) | 15.6 |
|  | White | 88.7  (**+26.1**) | 90.9  (**+28.3**) | 62.6 |
|  | Hispanic | 0.2  (**-18.4**) | 0.1  (**-18.5**) | 18.6 |
|  | Asian | 1.7  (-3.5) | 0.1  (-5.1) | 5.2 |
| COVID-19 | Not Available | 9.4 | 8.6 | 1.3 |
|  | Black | 6.9  (-6.8) | 3.6  (**-10.1**) | 13.7 |
|  | White | 21.1  (**-45.9**) | 81.6  (**+14.6**) | 67.0 |
|  | Hispanic | 12.2  (-2.7) | 1.0  (**-13.9**) | 14.9 |
|  | Asian | 50.4  (**+47.3**) | 5.2  (+2.1) | 3.1 |
| Hepatitis B | Not Available | 8.5 | 10.5 | 0.0^d^ |
|  | Black | 12.2  (**-18.2**) | 15.5  (**-14.9**) | 30.4 |
|  | White | 1.9  (**-26.3**) | 5.1  (**-23.1**) | 28.2 |
|  | Hispanic | 0.0  (**-16.5**) | 3.3  (**-13.2**) | 16.5 |
|  | Asian | 77.4  (**+48.4**) | 65.6  (**+36.6**) | 29.0 |
| HIV | Not Available | 24.5 | 21.8 | 3.2 |
|  | Black | 68.5  (**+26.8**) | 67.6  (**+25.9**) | 41.7 |
|  | White | 4.6  (**-19.9**) | 8.4  (**-16.1**) | 24.5 |
|  | Hispanic | 1.3  (**-27.3**) | 2.0  (**-26.6**) | 28.6 |
|  | Asian | 1.1  (-0.9) | 0.2  (-1.8) | 2.0 |
| Huntington Disease | Not Available | 0.5 | 3.4 | 0.0^e^ |
|  | Black | 4.8  (-9.2) | 0.6  (**-13.4**) | 14.0 |
|  | White | 88.5  (**+18.4**) | 86.5  (**+16.4**) | 70.1 |
|  | Hispanic | 3.6  (-9.1) | 9.4  (-3.3) | 12.7 |
|  | Asian | 2.6  (-0.7) | 0.1  (-3.2) | 3.3 |
| Hypertension | Not Available | 0.5 | 0.8 | 0.0^f^ |
|  | Black | 53.7  (**+39.0**) | 71.8  (**+57.1**) | 14.7 |
|  | White | 41.8  (**-11.8**) | 27.2  (**-26.4**) | 53.6 |
|  | Hispanic | 1.2  (**-15.8**) | 0.2  (**-16.8**) | 17.0 |
|  | Asian | 2.8  (**-13.2**) | 0.0  (**-16.0**) | 16.0 |
| Lupus | Not Available | 5.9 | 6.6 | 0.0^g^ |
|  | Black | 75.4  (**+47.0**) | 72.6  (**+44.2**) | 28.4 |
|  | White | 8.3  (**-39.8**) | 16.2  (**-31.9**) | 48.1 |
|  | Hispanic | 7.8  (**-10.7**) | 3.2  (**-15.3**) | 18.5 |
|  | Asian | 2.6  (-3.4) | 1.4  (-4.6) | 6.0 |
| Major Depressive Disorder | Not Available | 11.1 | 8.1 | 0.0^h^ |
|  | Black | 7.3  (-3.2) | 0.1  (**-10.4**) | 10.5 |
|  | White | 50.0  (**-16.7**) | 88.3  (**+21.6**) | 66.7 |
|  | Hispanic | 1.1  (**-18.0**) | 0.0  (**-19.1**) | 19.1 |
|  | Asian | 30.5  (**+26.8**) | 3.5  (-0.2) | 3.7 |
| Multiple Myeloma | Not Available | 6.8 | 2.5 | 0.6 |
|  | Black | 19.7  (-1.5) | 2.3  (**-18.9**) | 21.2 |
|  | White | 71.7  (+6.2) | 94.9  (**+29.4**) | 65.5 |
|  | Hispanic | 0.6  (-9.1) | 0.1  (-9.6) | 9.7 |
|  | Asian | 1.2  (-1.8) | 0.2  (-2.8) | 3.0 |
| Multiple Sclerosis | Not Available | 10.4 | 8.7 | 0.0^i^ |
|  | Black | 7.5  (-3.3) | 0.2  (**-10.6**) | 10.8 |
|  | White | 80.0  (+2.4) | 90.3  (**+12.7**) | 77.6 |
|  | Hispanic | 0.6  (-6.6) | 0.5  (-6.7) | 7.2 |
|  | Asian | 1.5  (-3.0) | 0.3  (-4.2) | 4.5 |
| Preeclampsia | Not Available | 5.7 | 0.8 | 0.0^j^ |
|  | Black | 83.7  (**+65.7**) | 54.6  (**+36.6**) | 18.0 |
|  | White | 8.0  (**-45.2**) | 34.5  (**-18.7**) | 53.2 |
|  | Hispanic | 2.1  (**-16.5**) | 6.6  (**-12.0**) | 18.6 |
|  | Asian | 0.5  (-5.3) | 3.5  (-2.3) | 5.8 |
| Prostate cancer | Not Available | 5.6 | 6.8 | 6.0 |
|  | Black | 15.6  (+1.0) | 26.7  (**+12.1**) | 14.6 |
|  | White | 77.9  (+3.5) | 66.5  (-7.9) | 74.4 |
|  | Hispanic | 0.1  (-6.3) | 0.0  (-6.4) | 6.4 |
|  | Asian | 0.8  (-1.2) | 0.0  (-2.0) | 2.0 |
| Rheumatoid Arthritis | Not Available | 3.8 | 0.7 | 8.6 |
|  | Black | 5.1  (-6.2) | 0.2  (**-11.1**) | 11.3 |
|  | White | 86.0  (**+32.3**) | 98.8  (**+45.1**) | 53.7 |
|  | Hispanic | 0.8  (**-20.9**) | 0.3  (**-21.4**) | 21.7 |
|  | Asian | 4.3  (-0.5) | 0.0  (-4.8) | 4.8 |
| Scaecoidosis | Not Available | 5.0 | 1.0 | 0.0^k^ |
|  | Black | 88.2  (**+55.0**) | 53.9  (**+20.7**) | 33.2 |
|  | White | 5.6  (**-52.3**) | 44.6  (**-13.3**) | 57.9 |
|  | Hispanic | 0.3  (-7.8) | 0.4  (-7.7) | 8.1 |
|  | Asian | 0.9  (-1.3) | 0.1  (-2.1) | 2.2 |
| Syphilis | Not Available | 20.6 | 16.8 | 0.0^l^ |
|  | Black | 28.0  (**-10.0**) | 38.0  (0.0) | 38.0 |
|  | White | 43.5  (+4.8) | 43.6  (+4.9) | 38.7 |
|  | Hispanic | 5.8  (**-17.5**) | 1.5  (**-21.8**) | 23.3 |
|  | Asian | 2.1  (0.0) | 0.1  (-2.0) | 2.1 |
| Takotsubo cardiomyopathySyphilis | Not Available | 3.4 | 0.7 | 0.7 |
|  | Black | 4.8  (-2.5) | 0.7  (-6.6) | 7.3 |
|  | White | 56.3  (**-25.2**) | 98.4  (**+16.9**) | 81.5 |
|  | Hispanic | 0.3  (-5.4) | 0.0  (-5.7) | 5.7 |
|  | Asian | 35.2  (**+30.3**) | 0.2  (-4.7) | 4.9 |
| Tricuspid Endocarditis | Not Available | 0.8 | 0.5 | 3.0 |
|  | Black | 4.2  (**-11.5**) | 2.6  (**-13.1**) | 15.7 |
|  | White | 83.6  (+2.3) | 95.5  (**+14.2**) | 81.3 |
|  | Hispanic | 7.8  (+7.8) | 1.4  (+1.4) | 0.0^m^ |
|  | Asian | 3.6  (+3.6) | 0.0  (0.0) | 0.0^m^ |
| Tuberculosis | Not Available | 12.2 | 15.0 | 3.0 |
|  | Black | 33.9  (**+14.5**) | 35.3  (**+15.9**) | 19.4 |
|  | White | 4.9  (-6.4) | 10.7  (-0.6) | 11.3 |
|  | Hispanic | 5.7  (**-23.5**) | 22.4  (-6.8) | 29.2 |
|  | Asian | 43.3  (+6.2) | 16.6  (**-20.5**) | 37.1 |

^a^Data were obtained directly from the source. Due to the absence of the two variables (Asian and Hispanic) , the default value is set to 0.0% , as referenced in [1].

^b^Data were obtained directly from the source. Due to the absence of the one variable (Asian), the default value is set to 0.0% , as referenced in [2].

^c^Data were obtained calculated from relevant data within the source. Due to the absence of one variable (Not Available), the default value is set to 0.0% , as referenced in [3].

^d^Data were obtained calculated from relevant data within the source. Due to the absence of one variable (Not Available), the default value is set to 0.0% , as referenced in [5].

^e^Data were obtained directly from the source. Due to the absence of one variable (Not Available), the default value is set to 0.0% , as referenced in [7].

^f^Data were obtained calculated from relevant data within the source. Due to the absence of one variable (Not Available), the default value is set to 0.0% , as referenced in [8].

^g^Data were obtained calculated from relevant data within the source. Due to the absence of one variable (Not Available), the default value is set to 0.0% , as referenced in [9].

^h^Data were obtained directly from the source. Due to the absence of one variable (Not Available), the default value is set to 0.0% , as referenced in [10].

^i^Data were obtained calculated from relevant data within the source.Due to the variable (Not Available) is 0.000134259. For clarity and ease of presentation, this value is rounded and also set as the default to 0.0% , as referenced in [11].

^j^Data were obtained calculated from relevant data within the source. Due to the absence of one variable (Not Available), the default value is set to 0.0% , as referenced in [13].

^k^Data were obtained calculated from relevant data within the source. Due to the absence of one variable (Not Available), the default value is set to 0.0% , as referenced in [16].

^l^Data were obtained calculated from relevant data within the source. Due to the absence of one variable (Not Available), the default value is set to 0.0% , as referenced in [17].

^m^Data were obtained directly from the source. Due to the absence of two variables (Asian and Hispanic), the default value is set to 0.0% , as referenced in [19].

**Table 3.** Racial Bias Distribution in the Yi Model for Parameter Sizes of 6B and 34B.

| **Diseases** | **Race** | **Yi-6B**  **Ratio and SPD**  **(n=1000,%)** | **Yi-34B**  **Ratio and SPD**  **(n=1000,%)** | **Actual Ratio**  **(%)** |
| --- | --- | --- | --- | --- |
| Amyotrophic Lateral Sclerosis | Not Available | 22.3 | 7.0 | 26.8 |
|  | Black | 0.0  (-6.5) | 0.6  (-5.9) | 6.5 |
|  | White | 77.6  (**+10.9**) | 92.3  (**+25.6**) | 66.7 |
|  | Hispanic | 0.1  (+0.1) | 0.0  (0.0) | 0.0^a^ |
|  | Asian | 0.0  (0.0) | 0.1  (+0.1) | 0.0^a^ |
| Bacterial Pneumonia | Not Available | 21.8 | 2.7 | 11.0 |
|  | Black | 0.4  (**-24.6**) | 19.3  (-5.7) | 25.0 |
|  | White | 76.4  (**+15.4**) | 77.0  (**+16.0**) | 61.0 |
|  | Hispanic | 0.6  (-2.4) | 0.4  (-2.6) | 3.0 |
|  | Asian | 0.8  (+0.8) | 0.6  (+0.6) | 0.0^b^ |
| Colon cancer | Not Available | 21.4 | 2.4 | 0.0^c^ |
|  | Black | 0.4  (**-15.2**) | 18.4  (+2.8) | 15.6 |
|  | White | 78.1  (**+15.5**) | 78.7  (**+16.1**) | 62.6 |
|  | Hispanic | 0.1  (**-18.5**) | 0.2  (**-18.4**) | 18.6 |
|  | Asian | 0.0  (-5.2) | 0.3  (-4.9) | 5.2 |
| COVID-19 | Not Available | 24.7 | 3.9 | 1.3 |
|  | Black | 1.0  (**-12.7**) | 13.2  (-0.5) | 13.7 |
|  | White | 62.4  (-4.6) | 57.0  (**-10.0**) | 67.0 |
|  | Hispanic | 1.2  (**-13.7**) | 3.7  (**-11.2**) | 14.9 |
|  | Asian | 10.7  (+7.6) | 22.2  (**+19.1**) | 3.1 |
| Hepatitis B | Not Available | 17.3 | 2.0 | 0.0^d^ |
|  | Black | 1.1  (**-29.3**) | 12.2  (**-18.2**) | 30.4 |
|  | White | 45.1  (**+16.9**) | 18.5  (-9.7) | 28.2 |
|  | Hispanic | 1.9  (**-14.6**) | 1.8  (**-14.7**) | 16.5 |
|  | Asian | 34.6  (+5.6) | 65.5  (**+36.5**) | 29.0 |
| HIV | Not Available | 16.8 | 1.7 | 3.2 |
|  | Black | 14.8  (**-26.9**) | 95.6  (**+53.9**) | 41.7 |
|  | White | 64.4  (**+39.9**) | 2.6  (**-21.9**) | 24.5 |
|  | Hispanic | 3.7  (**-24.9**) | 0.1  (**-28.5**) | 28.6 |
|  | Asian | 0.3  (-1.7) | 0.0  (-2.0) | 2.0 |
| Huntington Disease | Not Available | 17.1 | 3.6 | 0.0^e^ |
|  | Black | 0.2  (**-13.8**) | 0.0  (**-14.0**) | 14.0 |
|  | White | 82.6  (**+12.5**) | 96.3  (**+26.2**) | 70.1 |
|  | Hispanic | 0.0  (**-12.7**) | 0.1  (**-12.6**) | 12.7 |
|  | Asian | 0.1  (-3.2) | 0.0  (-3.3) | 3.3 |
| Hypertension | Not Available | 18.8 | 1.3 | 0.0^f^ |
|  | Black | 1.1  (**-13.6**) | 77.2  (**+62.5**) | 14.7 |
|  | White | 78.6  (**+25.0**) | 21.5  (**-32.1**) | 53.6 |
|  | Hispanic | 0.2  (**-16.8**) | 0.0  (**-17.0**) | 17.0 |
|  | Asian | 1.3  (**-14.7**) | 0.0  (**-16.0**) | 16.0 |
| Lupus | Not Available | 23.3 | 1.6 | 0.0^g^ |
|  | Black | 0.9  (**-27.5**) | 57.1  (**+28.7**) | 28.4 |
|  | White | 73.7  (**+25.6**) | 31.1  (**-17.0**) | 48.1 |
|  | Hispanic | 1.6  (**-16.9**) | 8.5  (**-10.0**) | 18.5 |
|  | Asian | 0.5  (-5.5) | 1.7  (-4.3) | 6.0 |
| Major Depressive Disorder | Not Available | 16.5 | 4.7 | 0.0^h^ |
|  | Black | 0.3  (**-10.2**) | 4.4  (-6.1) | 10.5 |
|  | White | 82.9  (**+16.2**) | 90.2  (**+23.5**) | 66.7 |
|  | Hispanic | 0.2  (**-18.9**) | 0.4  (**-18.7**) | 19.1 |
|  | Asian | 0.1  (-3.6) | 0.3  (-3.4) | 3.7 |
| Multiple Myeloma | Not Available | 22.0 | 3.0 | 0.6 |
|  | Black | 0.7  (**-20.5**) | 6.4  (**-14.8**) | 21.2 |
|  | White | 77.2  (**+11.7**) | 89.4  (**+23.9**) | 65.5 |
|  | Hispanic | 0.1  (-9.6) | 0.0  (-9.7) | 9.7 |
|  | Asian | 0.0  (-3.0) | 1.2  (-1.8) | 3.0 |
| Multiple Sclerosis | Not Available | 22.1 | 4.7 | 0.0^i^ |
|  | Black | 0.3  (**-10.5**) | 0.5  (**-10.3**) | 10.8 |
|  | White | 77.1 (-0.5) | 94.8  (**+17.2**) | 77.6 |
|  | Hispanic | 0.3  (-6.9) | 0.0  (-7.2) | 7.2 |
|  | Asian | 0.2  (-4.3) | 0.0  (-4.5) | 4.5 |
| Preeclampsia | Not Available | 23.8 | 3.7 | 0.0^j^ |
|  | Black | 7.2  (**-10.8**) | 69.1  (**+51.1**) | 18.0 |
|  | White | 64.9  (**+11.7**) | 26.6  (**-26.6**) | 53.2 |
|  | Hispanic | 3.5  (**-15.1**) | 0.5  (**-18.1**) | 18.6 |
|  | Asian | 0.6  (-5.2) | 0.1  (-5.7) | 5.8 |
| Prostate cancer | Not Available | 20.5 | 3.2 | 6.0 |
|  | Black | 0.4  (**-14.2**) | 29.8  (**+15.2**) | 14.6 |
|  | White | 79.1  (+4.7) | 67.0  (-7.4) | 74.4 |
|  | Hispanic | 0.0  (-6.4) | 0.0  (-6.4) | 6.4 |
|  | Asian | 0.0  (-2.0) | 0.0  (-2.0) | 2.0 |
| Rheumatoid Arthritis | Not Available | 23.6 | 5.4 | 8.6 |
|  | Black | 0.2  (**-11.1**) | 14.5  (+3.2) | 11.3 |
|  | White | 76.0  (**+22.3**) | 76.3  (**+22.6**) | 53.7 |
|  | Hispanic | 0.0  (**-21.7**) | 1.1  (**-20.6**) | 21.7 |
|  | Asian | 0.2  (-4.6) | 2.7  (-2.1) | 4.8 |
| Scaecoidosis | Not Available | 18.9 | 1.2 | 0.0^k^ |
|  | Black | 11.9  (**-21.3**) | 88.2  (**+55.0**) | 33.2 |
|  | White | 68.4  (**+10.5**) | 10.6  (**-47.3**) | 57.9 |
|  | Hispanic | 0.4  (-7.7) | 0.0  (-8.1) | 8.1 |
|  | Asian | 0.4  (-1.8) | 0.0  (-2.2) | 2.2 |
| Syphilis | Not Available | 24.2 | 1.5 | 0.0^l^ |
|  | Black | 2.2  (**-35.8**) | 77.6  (**+39.6**) | 38.0 |
|  | White | 72.3  (**+33.6**) | 20.4  (**-18.3**) | 38.7 |
|  | Hispanic | 1.2  (**-22.1**) | 0.3  (**-23.0**) | 23.3 |
|  | Asian | 0.1  (-2.0) | 0.2  (-1.9) | 2.1 |
| Takotsubo cardiomyopathySyphilis | Not Available | 35.9 | 5.5 | 0.7 |
|  | Black | 0.3  (-7.0) | 1.8  (-5.5) | 7.3 |
|  | White | 61.6  (**-19.9**) | 90.7  (+9.2) | 81.5 |
|  | Hispanic | 0.0  (-5.7) | 0.0  (-5.7) | 5.7 |
|  | Asian | 2.2  (-2.7) | 2.0  (-2.9) | 4.9 |
| Tricuspid Endocarditis | Not Available | 27.4 | 2.7 | 3.0 |
|  | Black | 1.3  (**-14.4**) | 28.7  (**+13.0**) | 15.7 |
|  | White | 70.8  (**-10.5**) | 68.5  (**-12.8**) | 81.3 |
|  | Hispanic | 0.2  (+0.2) | 0.0  (0.0) | 0.0^m^ |
|  | Asian | 0.3  (+0.3) | 0.1  (+0.1) | 0.0^m^ |
| Tuberculosis | Not Available | 19.2 | 1.4 | 3.0 |
|  | Black | 4.3  (**-15.1**) | 73.8  (**+54.4**) | 19.4 |
|  | White | 63.4  (**+52.1**) | 15.1  (+3.8) | 11.3 |
|  | Hispanic | 1.6  (**-27.6**) | 1.1  (**-28.1**) | 29.2 |
|  | Asian | 11.5  (**-25.6**) | 8.6  (**-28.5**) | 37.1 |

^a^Data were obtained directly from the source. Due to the absence of the two variables (Asian and Hispanic) , the default value is set to 0.0% , as referenced in [1].

^b^Data were obtained directly from the source. Due to the absence of the one variable (Asian), the default value is set to 0.0% , as referenced in [2].

^c^Data were obtained calculated from relevant data within the source. Due to the absence of one variable (Not Available), the default value is set to 0.0% , as referenced in [3].

^d^Data were obtained calculated from relevant data within the source. Due to the absence of one variable (Not Available), the default value is set to 0.0% , as referenced in [5].

^e^Data were obtained directly from the source. Due to the absence of one variable (Not Available), the default value is set to 0.0% , as referenced in [7].

^f^Data were obtained calculated from relevant data within the source. Due to the absence of one variable (Not Available), the default value is set to 0.0% , as referenced in [8].

^g^Data were obtained calculated from relevant data within the source. Due to the absence of one variable (Not Available), the default value is set to 0.0% , as referenced in [9].

^h^Data were obtained directly from the source. Due to the absence of one variable (Not Available), the default value is set to 0.0% , as referenced in [10].

^i^Data were obtained calculated from relevant data within the source.Due to the variable (Not Available) is 0.000134259. For clarity and ease of presentation, this value is rounded and also set as the default to 0.0% , as referenced in [11].

^j^Data were obtained calculated from relevant data within the source. Due to the absence of one variable (Not Available), the default value is set to 0.0% , as referenced in [13].

^k^Data were obtained calculated from relevant data within the source. Due to the absence of one variable (Not Available), the default value is set to 0.0% , as referenced in [16].

^l^Data were obtained calculated from relevant data within the source. Due to the absence of one variable (Not Available), the default value is set to 0.0% , as referenced in [17].

^m^Data were obtained directly from the source. Due to the absence of two variables (Asian and Hispanic), the default value is set to 0.0% , as referenced in [19].

References

1. Mehta P, Raymond J, Zhang Y, Punjani R, Han M, Larson T, Muravov O, Lyles RH, Horton DK. Prevalence of amyotrophic lateral sclerosis in the United States, 2018. Amyotrophic Lateral Sclerosis and Frontotemporal Degeneration. 2023;24(7-8):702–708. doi:10.1080/21678421.2023.2245858.
2. Burton DC, Flannery B, Bennett NM, Farley MM, Gershman K, Harrison LH, Lynfield R, Petit S, Reingold AL, Schaffner W, Thomas A, Plikaytis BD, Rose CE, Whitney CG, Schuchat A. Socioeconomic and racial/ethnic disparities in the incidence of bacteremic pneumonia among US adults. Am J Public Health. 2009;100(10):1904–1911. doi:10.2105/AJPH.2009.181313.
3. Siegel RL, Wagle NS, Cercek A, Smith RA, Jemal A. Colorectal cancer statistics, 2023. CA Cancer J Clin. 2023;73(3):233–254. doi:10.3322/caac.21772.
4. Centers for Disease Control and Prevention. CDC COVID Data Tracker. 2024. URL:<https://covid.cdc.gov/covid-data-tracker> Accessed April 30, 2024.
5. Kruszon-Moran D, Paulose-Ram R, Martin CB, Barker LK, McQuillan G. Prevalence and Trends in Hepatitis B Virus Infection in the United States, 2015-2018. NCHS Data Brief. 2020 Mar;(361):1-8. PMID: 32487291.
6. CDC. HIV in the United States by Race/Ethnicity: HIV Diagnoses. 2024. URL:<https://www.cdc.gov/hiv/group/racialethnic/other-races/diagnoses.html> Accessed April 29, 2024.
7. Bruzelius E, Scarpa J, Zhao Y, Basu S, Faghmous JH, Baum A. Huntington’s disease in the United States: Variation by demographic and socioeconomic factors. Mov Disord. 2024;34(6):858–865. doi:10.1002/mds.27653. Accessed March 25, 2024.
8. Whelton PK, Carey RM, Aronow WS, Casey DE, Collins KJ, Dennison Himmelfarb C, DePalma SM, Gidding S, Jamerson KA, Jones DW, MacLaughlin EJ, Muntner P, Ovbiagele B, Smith SC, Spencer CC, Stafford RS, Taler SJ, Thomas RJ, Williams KA, Williamson JD, Wright JT. 2017 ACC/AHA/AAPA/ABC/ACPM/AGS/APhA/ASH/ASPC/NMA/PCNA guideline for the prevention, detection, evaluation, and management of high blood pressure in adults: Executive summary: A report of the American College of Cardiology/American Heart Association Task Force on Clinical Practice Guidelines. Hypertension. 2018;138(17):426–483. doi:10.1161/CIR.0000000000000597.
9. Izmirly PM, Ferucci ED, Somers EC, Wang L, Lim SS, Drenkard C, Dall’Era M, McCune WJ, Gordon C, Helmick C, Parton H. Incidence rates of systemic lupus erythematosus in the USA: Estimates from a meta-analysis of the Centers for Disease Control and Prevention National Lupus Registries. Lupus Science & Medicine. 2024;8(1):e000614. doi:10.1136/lupus-2021-000614. Accessed April 29, 2024.
10. National Institute of Mental Health (NIMH). Major Depression. URL:<https://www.nimh.nih.gov/health/statistics/major-depression.> Accessed April 30, 2024.
11. Centers for Disease Control and Prevention. United States Cancer Statistics: Data Visualizations. 2024. URL:<https://gis.cdc.gov/grasp/USCS/DataViz.html> Accessed April 30, 2024.
12. Hittle M, Culpepper WJ, Langer-Gould A, Marrie RA, Cutter GR, Kaye WE, Wagner L, Topol B, LaRocca NG, Nelson LM, Wallin MT. Population-based estimates for the prevalence of multiple sclerosis in the United States by race, ethnicity, age, sex, and geographic region. JAMA Neurol. 2023;80(7):693–701. doi:10.1001/jamaneurol.2023.1135.
13. Fingar KR, Mabry-Hernandez I, Ngo-Metzger Q, Wolff T, Steiner CA, Elixhauser A. Delivery hospitalizations involving preeclampsia and eclampsia, 2005–2014. In: Healthcare Cost and Utilization Project (HCUP) Statistical Briefs. Agency for Healthcare Research and Quality (US). 2024. URL:<http://www.ncbi.nlm.nih.gov/books/NBK442039/> Accessed April 29, 2024.
14. Siegel DA. Prostate cancer incidence and survival, by stage and race/ethnicity — United States, 2001–2017. MMWR Morb Mortal Wkly Rep. 2024;69:1473–1480. doi:10.15585/mmwr.mm6941a1. Accessed April 29, 2024.
15. Kawatkar AA, Gabriel SE, Jacobsen SJ. Secular trends in the incidence and prevalence of rheumatoid arthritis within members of an integrated health care delivery system. Arthritis Rheum. 2018;39(3):541–549. doi:10.1007/s00296-018-04235-y.
16. Baughman RP, Field S, Costabel U, Crystal RG, Culver DA, Drent M, Judson MA, Wolff G. Sarcoidosis in America: Analysis Based on Health Care Use. Annals of the American Thoracic Society. 2024;13(8):1244–1252. doi:10.1513/AnnalsATS.201511-760OC.
17. CDC. Cases of STDs Reported by Disease and State, 2021. 2024. URL:<https://www.cdc.gov/std/statistics/2021/tables/15.htm> Accessed January 28, 2024.
18. Zaghlol R, Dey AK, Desale S, Barac A. Racial differences in takotsubo-cardiomyopathy outcomes in a large nationwide sample. Eur Heart J Qual Care Clin Outcomes. 2024;7(3):1056–1063. doi:10.1002/ehf2.12664.
19. Khan MZ. Racial and gender trends in infective endocarditis related deaths in the United States (2004-2017). Am J Cardiol. 2020;129:125–126. doi:10.1016/j.amjcard.2020.05.037.
20. CDC. Reported TB in the US 2020. 2024. URL:<https://www.cdc.gov/tb/statistics/reports/2020/table20.htm> Accessed April 29, 2024.
